# Genetic Deletion of the Desmosomal Component *Desmoplakin* Promotes Tumor Microinvasion in a Mouse Model of Pancreatic Neuroendocrine Carcinogenesis

**DOI:** 10.1371/journal.pgen.1001120

**Published:** 2010-09-16

**Authors:** Matthew G. H. Chun, Douglas Hanahan

**Affiliations:** 1 Department of Biochemistry and Biophysics, Helen Diller Family Comprehensive Cancer Center, and Diabetes Center, University of California San Francisco, San Francisco, California, United States of America; 2 Program in Biological Sciences, University of California San Francisco, San Francisco, California, United States of America; 3 Swiss Institute for Experimental Cancer Research (ISREC), Swiss Federal Institute of Technology Lausanne (EPFL), Lausanne, Switzerland; Fred Hutchinson Cancer Research Center, United States of America

## Abstract

We used the *RIP1-Tag2* (*RT2*) mouse model of islet cell carcinogenesis to profile the transcriptome of pancreatic neuroendocrine tumors (PNET) that were either non-invasive or highly invasive, seeking to identify pro- and anti-invasive molecules. Expression of multiple components of desmosomes, structures that help maintain cellular adhesion, was significantly reduced in invasive carcinomas. Genetic deletion of one of these desmosomal components, *desmoplakin*, resulted in increased local tumor invasion without affecting tumor growth parameters in *RT2* PNETs. Expression of *cadherin 1*, a component of the adherens junction adhesion complex, was maintained in these tumors despite the genetic deletion of *desmoplakin*. Our results demonstrate that loss of *desmoplakin* expression and resultant disruption of desmosomal adhesion can promote increased local tumor invasion independent of adherens junction status.

## Author Summary

The ability of a tumor to invade into the surrounding normal tissue is one hallmark of a malignant cancer. We sought to identify factors that either restrict or promote tumor invasion in a genetically engineered mouse model of pancreatic neuroendocrine cancer by characterizing the transcriptional profiles of the non-invasive and invasive pancreatic neuroendocrine tumors (PNET) that develop in this model. This analysis demonstrated that multiple genes encoding components of desmosomes, cellular structures dedicated to the maintenance of cell-cell adhesion, were expressed at much lower levels in invasive PNETs, suggesting that loss of desmosomal adhesion contributes to the development of an invasive phenotype in these tumors. Genetic deletion of one of these desmosomal components in PNET-bearing mice resulted in increased local tumor invasion. These results are important since the development of an invasive phenotype is associated with a poor prognosis for many human cancers and is often a precursor to the development of distant metastases. Our findings demonstrate one mechanism by which tumors can acquire such an invasive phenotype and may prove useful in evaluating the malignancy of human cancers.

## Introduction

The ability of a tumor to invade into the surrounding normal tissue marks a critical step in the transition from benign to malignant tumor growth. The acquisition of this hallmark of cancer is associated with poor prognosis for many human cancers and is often considered a precursor to the development of metastases [1]. As such, considerable effort has been directed towards identifying invasion promoting and suppressing molecules and the mechanisms by which they modulate a tumor's invasive phenotype [2].

Amongst the discernible barriers to the acquisition of an invasive growth phenotype is cell-cell adhesion, and cellular alterations that result in disrupted, reduced, or otherwise functionally altered cellular adhesion are strongly associated with the progression to a malignant tumor phenotype [3]–[5]. The importance of sustaining cellular adhesion for homeostasis, particularly in epithelial tissues, is evident in the number of distinct structures whose primary function is to maintain cell-cell interconnections, which include the adherens junctions (AJs), desmosomes, and tight junctions [6], [7]. These complexes share many structural similarities, including the presence of transmembrane proteins – typified by the cadherins – that mediate adhesive connections with neighboring cells as well as intracellular molecules – exemplified by the catenin and the plakin families – that connect these transmembrane components to the cytoskeleton [6], [7]. In particular, changes in the expression and/or function of AJ components have been associated with malignant cancers, and numerous studies have focused on the role of AJs in restricting invasive growth [3], [8], [9].

In this study, we utilized the *RIP1-Tag2* (*RT2*) mouse model of cancer to identify the mechanisms by which tumors acquire invasive growth capabilities. *RT2* mice develop multiple pancreatic neuroendocrine tumors (PNET) by 12–14 weeks of age due to the expression of the SV40 T antigen oncoprotein (*Tag*) in the pancreatic β cells [10]. This model has proven useful in characterizing many aspects of tumorigenesis due to its relatively synchronous and predictable progression through distinctive lesional stages that culminate in invasive carcinomas [11]–[13]. We used this model to identify pro- and anti-invasive molecules in an unbiased fashion by comparing the non-invasive islet tumors to highly invasive carcinomas using microarray profiling of the mRNA transcriptome. We identified several components of desmosomes whose expression was significantly decreased in invasive tumors, implicating attenuation of desmosomal function in malignant progression. To assess this hypothesis, we engineered into the oncogene-expressing cancer cells in *RT2* mice a genetic deletion of *desmoplakin* (*Dsp*; MGI: 109611), an intracellular protein critical for desmosomal stability [14]. Loss of *Dsp* led to an increased incidence of invasive carcinomas providing strong evidence that desmosomal adhesion acts as a distinct barrier to invasive tumor growth.

## Results

### Expression of desmosomal components is lost in invasive *RT2* tumor lesions

We chose to use the *RT2* mouse model of cancer to characterize mechanisms governing the switch from benign to invasive tumor growth since a broad spectrum of invasive tumor lesions develop in end-stage *RT2* animals. These include the non-invasive islet tumor (IT), the focally invasive carcinoma type-1 (IC1), and the broadly invasive carcinoma type-2 (IC2) [15].

To evaluate potential mechanisms regulating invasive tumor growth in this model, we isolated tissue from IT and IC2 lesions in end-stage *RT2* animals by laser capture microdissection and then profiled the mRNA transcriptome. The IC2 class showed widespread transcriptional changes as compared to the IT class (Dataset S1). We chose to focus our attention on differentially expressed genes encoding components of two cell-cell adhesion structures, namely adherens junctions and desmosomes (Table 1), since elements of each were prominently downregulated. The expression of *cadherin 1* (*Cdh1*, also known as *E-cadherin*; MGI: 88354), a molecule previously demonstrated to restrict invasive growth in this and other models [8], [16], was decreased in IC2 lesions as expected. Interestingly, *Cdh1* was the only member of AJs that was significantly altered in IC2 lesions (Table 1). In contrast, multiple genes encoding components of desmosomes were significantly reduced in IC2 lesions (Table 1). Moreover, the expression of several desmosomal genes in addition to *Cdh1* was progressively reduced in the distinctive stages of PNET tumorigenesis in *RT2* mice as well as in human PNETs as compared to normal human pancreatic islets, when total lesional stages, in particular ungraded tumors, were analyzed (Figure S1) [13]. Although the expression of these genes was reduced in ungraded whole tumors in comparison to normal islets, their levels were further reduced in the microdissected invasive IC2 lesions (Table 1). Based on these results, we sought to determine what role desmosomal adhesion might play in regulating invasive tumor growth in this mouse model of cancer.

**Table 1:** Summary of Microarray Results for Components of Desmosomes and Adherens Junctions.

P-value	Fold-change^a^	Affymetrix probe ID^b^	Gene symbol	Gene name	Synonyms	Complex
0.017	−7.81	1426911_at	*Dsc2*	*Desmocollin 2*		Desmosome
0.027	−7.52	1439476_at	*Dsg2*	*Desmoglein 2*		Desmosome
0.053	−12.61	1435494_s_at	*Dsp*	*Desmoplakin*		Desmosome
0.019	−14.03	1449799_s_at	*Pkp2*	*Plakophilin 2*		Desmosome
0.029	−12.99	1448261_at	*Cdh1*	*Cadherin 1*	*E-cadherin*	Adherens junction
0.181	−1.33	1437807_x_at	*Ctnna1*	*Catenin alpha 1*	*Alpha E catenin*	Adherens junction
0.795	−1.09	1430533_a_at	*Ctnnb1*	*Catenin beta 1*	*Beta catenin*	Adherens junction
0.444	1.29	1445830_at	*Ctnnd1*	*Catenin delta 1*	*P120 catenin*	Adherens junction
0.852	1.30	1426873_s_at	*Jup*	*Junction plakoglobin*	*Gamma catenin*	Adherens junction; desmosome

a Fold-change represents the widely invasive carcinoma type-2 (IC2) class as compared to the non-invasive islet tumor (IT) class of *RIP1-Tag2* tumor lesions.

b Affymetrix probe ID corresponds to the Mouse Genome 430 2.0 Array.

**Figure 1:**
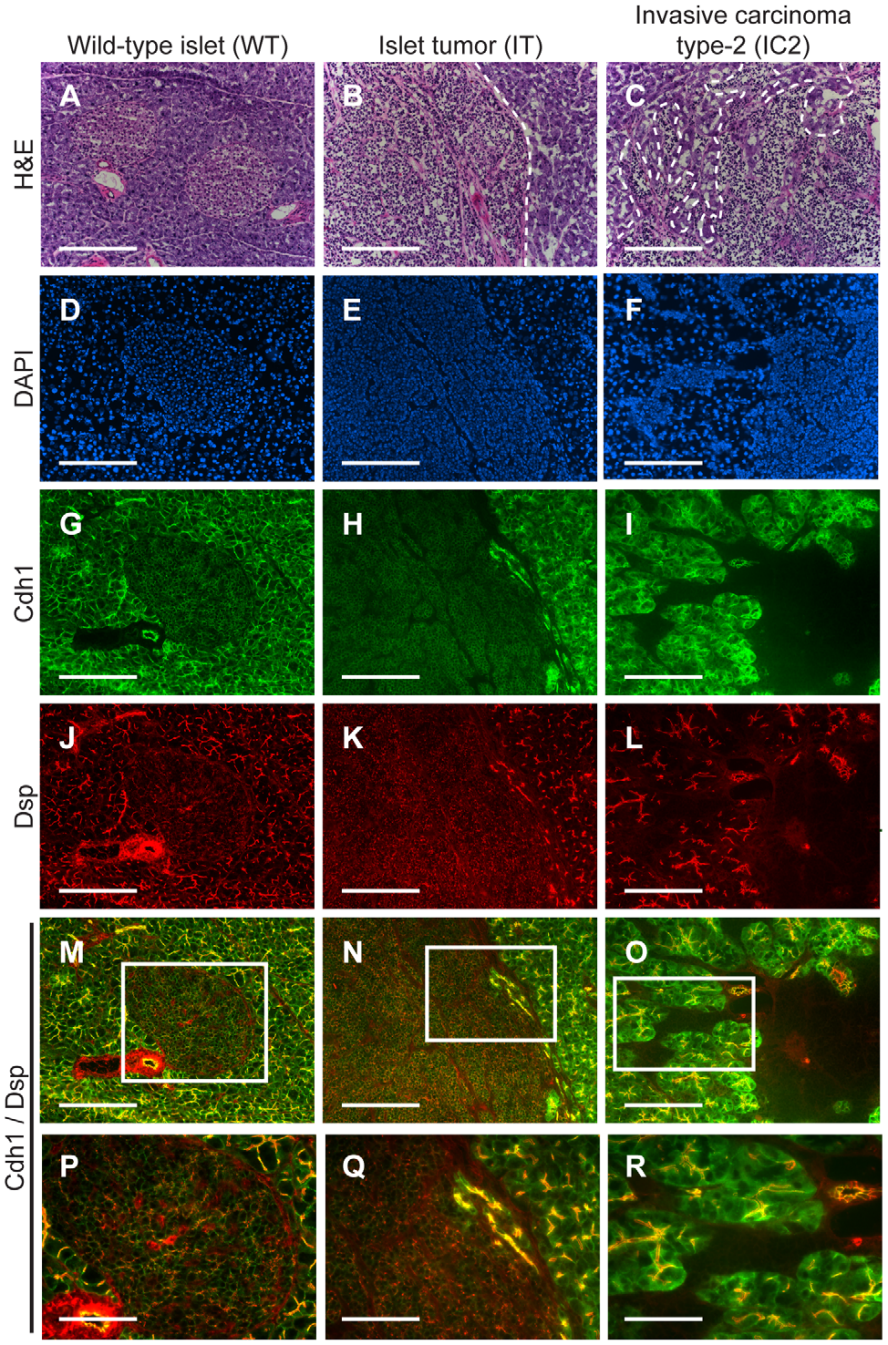
*Desmoplakin* and *cadherin 1* expression in wild-type islets and *RT2* PNETs. Expression of *desmoplakin* (*Dsp*) and *cadherin 1* (*Cdh1*) is lost in the IC2 but not the IT grade of PNET in *RT2* mice. (A–C) H&E staining of a normal islet from a wild-type *B6* mouse and of an IT and an IC2 tumor from an end-stage *RT2* mouse. Dashed lines demarcate tumor margins. (D–F) Immunofluorescence staining with DAPI to visualize cellularity. (G–I) Immunofluorescence staining for *Cdh1*. (J–L) Immunofluorescence staining for *Dsp*. (M–O) Merge of *Cdh1* and *Dsp* immunofluorescence staining (G–L). (P–R) Higher magnification of the boxed regions in M–O. Scale bars represent 200 µm (A–O) and 100 µm (P–R).

To confirm the microarray results, we performed immunohistochemistry for multiple desmosomal components. Staining for *Dsp* and for one of the desmosomal cadherins, *desmoglein 2* (*Dsg2*; MGI: 1196466), as well as for *Cdh1* demonstrated that these molecules are expressed in the pancreatic islets as well as in the pancreatic ducts and the exocrine pancreas of wild-type animals (Figure 1 and Figure S2). In tumors of end-stage *RT2* animals, the expression of all three molecules was maintained in IT lesions and was largely extinguished in IC2 lesions (Figure 1 and Figure S2). In contrast to *Cdh1*, expression of *catenin beta 1* (*Ctnnb1*; MGI: 88276), another component of AJs, was maintained in both IT and IC2 lesions, comparable to wild-type islets (Figure S3). This result is consistent both with the microarray result demonstrating that *Cdh1* was the only AJ component to show any change in expression and with a previous study suggesting that *Ctnnb1* does not contribute to *RT2* tumorigenesis [17]. Collectively, these data confirm the microarray results and suggest the hypothesis that loss of desmosomal adhesion might contribute to the development of an invasive phenotype.

### β cell specific deletion of *Dsp* in *RT2* animals

To address the hypothesis raised by the microarray and immunohistochemistry results, we asked whether functionally disrupting desmosomal activity *in vivo* would promote invasive tumor growth in *RT2* mice. To accomplish this, we chose to genetically delete *Dsp* since there is a single *Dsp* gene as compared to other components of desmosomes for which there are multiple non-allelic genes [6]. Furthermore, ablation of *Dsp in vivo* has previously been shown to impair desmosome function [14]. Since the *Dsp* whole body knockout is embryonic lethal [14], we employed the *Cre/loxP* system to ablate the *Dsp* gene specifically in the pancreatic β cells, the same cells that express the *Tag* oncogene in *RT2* mice. In combination with a *Dsp^Flox^* allele [18], we used a mouse line in which a tamoxifen-regulatable *Cre* recombinase is controlled by the *pancreatic duodenal homeobox gene 1* promoter (*Pdx1-Cre^ER^*) [19]. *Pdx1* is expressed in all pancreatic lineages during development and is variably expressed in the adult pancreas, in particular being widely expressed in β cells [20], [21].

We intercrossed *RT2+*; *Dsp^Flox/WT^* with *Pdx1-Cre^ER^+*; *Dsp^Flox/WT^* mice to generate the appropriate genotypes, and all expected genotypes and genders were observed in approximate Mendellian ratios (Table S1 and Table S2). To induce *Cre* activity, all *Pdx1-Cre^ER^* positive mice were given tamoxifen for five consecutive days beginning at 10 weeks of age when incipient tumors are first observed in *RT2* mice [22]. In the absence of the *RT2* transgene, genetic ablation of *Dsp* resulted in uniform loss of *Dsp* expression in the pancreatic islets, as determined by immunohistochemistry (Figure S4). Deletion of *Dsp* did not cause any change in *Cdh1* expression or in the gross morphological appearance of the non-oncogene-expressing islets (Figure S4). Loss of *Dsp* was accompanied by significantly reduced *Dsg2* expression in the pancreatic islets whereas the expression of *insulin* (*Ins*), the hormone produced by β cells, did not appear to be affected (Figure S5). These results are consistent with compromised desmosomal adhesion, although we cannot strictly rule out the possibility that some residual desmosomal function persists in the absence of *Dsp*. Ablation of *Dsp* in normal pancreatic islets did not affect multiple physiological parameters, such as body mass and fasting glucose levels, and its expression in this tissue compartment is apparently dispensable in adult mice (Figure S6), setting the stage to assess the impact of its loss on PNETs arising from such islets. Lastly, the tamoxifen induction regimen by itself had no obvious effect on any aspect of *RT2* tumorigenesis examined, including tumor invasion, when tamoxifen was applied to *RT2* mice that lacked the *Pdx1-Cre^ER^* and *Dsp^Flox^* alleles (Figure S7).

### Loss of *Dsp* does not affect tumor growth parameters in *RT2* mice

**Figure 2:**
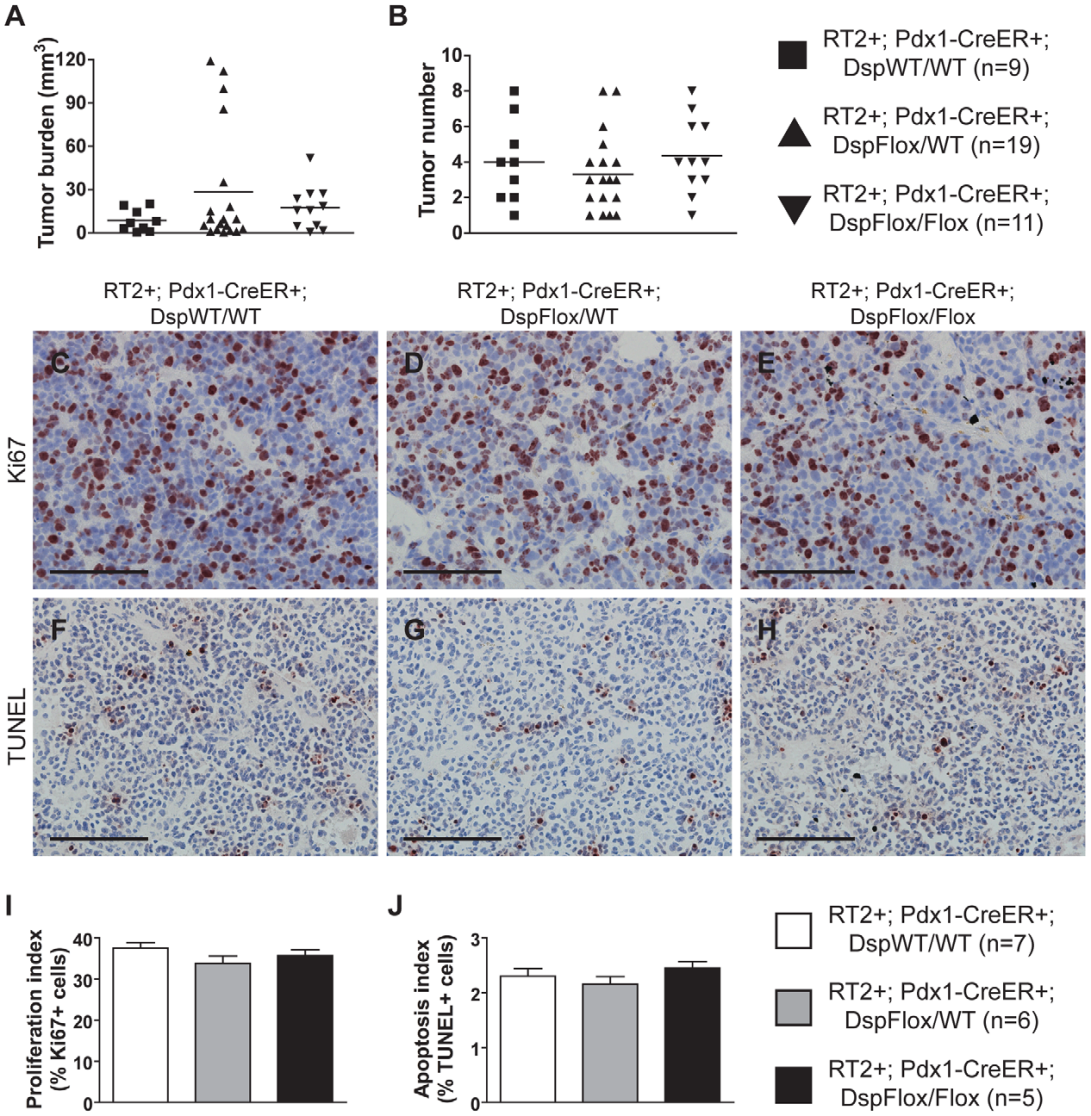
Genetic deletion of *desmoplakin* does not affect tumor growth parameters in *RT2* PNETs. Conditional genetic deletion of *Dsp* in angiogenic islet dysplasias and incipient solid tumors does not affect tumor formation or tumor growth parameters in *RT2* mice. (A–B) Tumor burden and tumor number in *RT2+*; *Pdx1-Cre^ER^+*; *Dsp^WT/WT^*, *RT2+*; *Pdx1-Cre^ER^+*; *Dsp^Flox/WT^*, and *RT2+*; *Pdx1-Cre^ER^+*; *Dsp^Flox/Flox^* mice. *Cre* activity was induced at 10 weeks, and mice were sacrificed at 14 weeks. Data shown are individual values plus mean. Groups are not statistically different for these metrics. (C–E) Ki67 staining on tumors from *RT2+*; *Pdx1-Cre^ER^+*; *Dsp^WT/WT^*, *RT2+*; *Pdx1-Cre^ER^+*; *Dsp^Flox/WT^*, and *RT2+*; *Pdx1-Cre^ER^+*; *Dsp^Flox/Flox^* mice. (F–H) TUNEL staining on tumors from *RT2+*; *Pdx1-Cre^ER^+*; *Dsp^WT/WT^*, *RT2+*; *Pdx1-Cre^ER^+*; *Dsp^Flox/WT^*, and *RT2+*; *Pdx1-Cre^ER^+*; *Dsp^Flox/Flox^* mice. (I) Quantification of C–E. Data shown are mean plus standard error. Groups are not statistically different. (J) Quantification of F–H. Data shown are mean plus standard error. Groups are not statistically different. Scale bars represent 100 µm (C–H).

Induced loss of *Dsp* at 10 weeks of age did not affect any of the tumor growth parameters in *RT2* mice that were sacrificed 4 weeks later. No significant changes were observed in the number of tumors that developed nor in the collective tumor burden when comparing *RT2+*; *Pdx1-Cre^ER^+*; *Dsp^Flox/Flox^* mice and littermate controls (Figure 2A–B). Furthermore, the rates of tumor proliferation and tumor apoptosis, as judged by the levels of the proliferation marker Ki67 and the TUNEL assay respectively, were indistinguishable between groups (Figure 2C–J). Thus, we conclude that the loss of *Dsp* does not affect tumor growth in this model.

### Loss of *Dsp* leads to increased local tumor invasion in *RT2* mice

**Figure 3:**
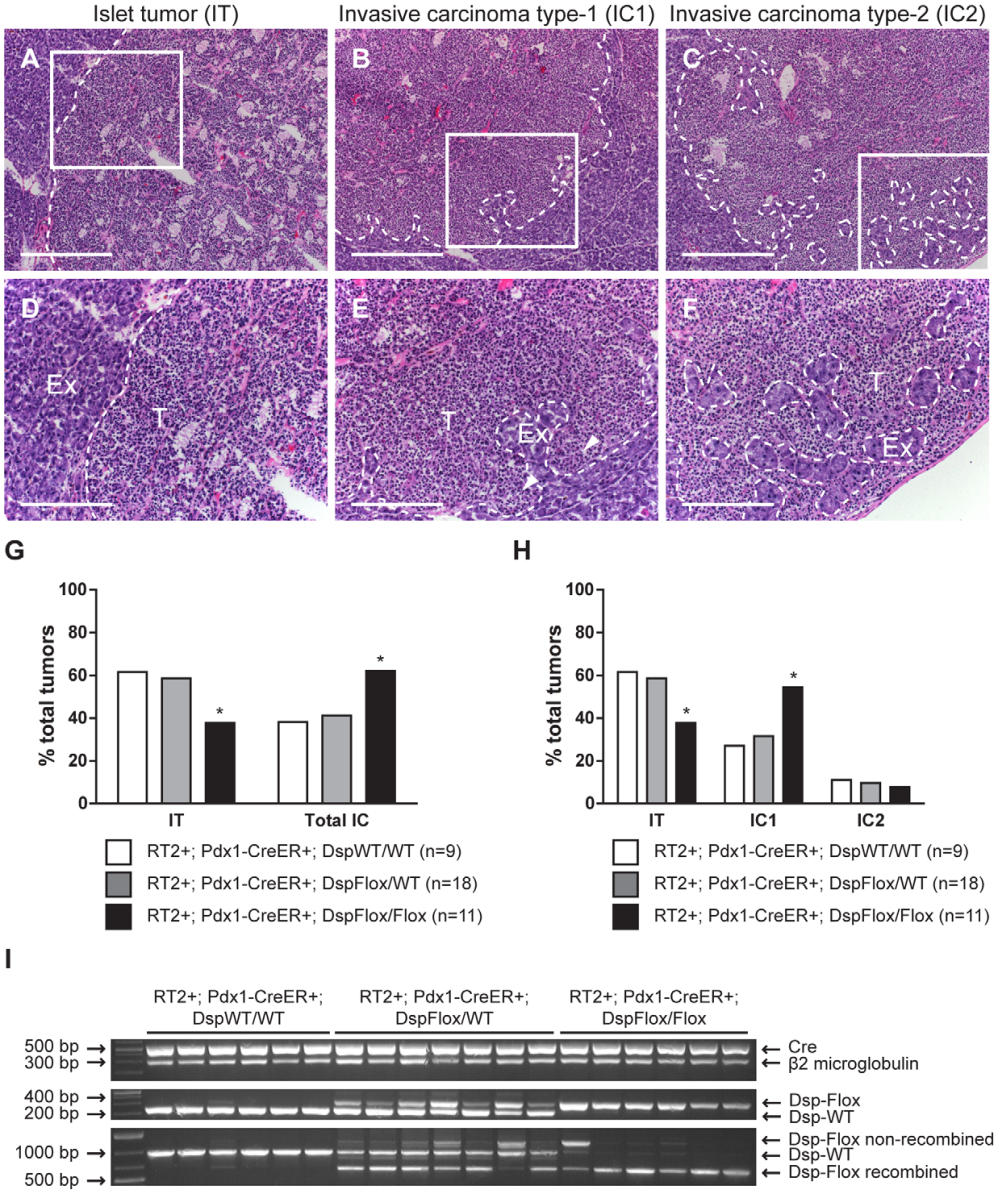
Genetic deletion of *desmoplakin* leads to increased local tumor invasion in *RT2* mice. Conditional genetic deletion of *Dsp* in angiogenic islet dysplasias and incipient solid tumors increases the rate of progression to focally invasive IC1 tumors in *RT2* mice. (A–C) H&E staining of a non-invasive IT tumor lesion, a focally invasive IC1 tumor lesion, and a broadly invasive IC2 tumor lesion from *RT2+*; *Pdx1-Cre^ER^+*; *Dsp^WT/WT^*, *RT2+*; *Pdx1-Cre^ER^+*; *Dsp^Flox/WT^*, and *RT2+*; *Pdx1-Cre^ER^+*; *Dsp^Flox/Flox^* mice. (D–F) Higher magnification of the boxed regions in A–C. T indicates tumor region and Ex indicates exocrine pancreas. Dashed lines demarcate tumor margins. Arrowheads indicate regions of tumor invasion. (G) Quantification of tumor invasiveness represented as the percentage of IT lesions or total IC lesions (IC1+IC2) in *RT2+*; *Pdx1-Cre^ER^+*; *Dsp^WT/WT^*, *RT2+*; *Pdx1-Cre^ER^+*; *Dsp^Flox/WT^*, and *RT2+*; *Pdx1-Cre^ER^+*; *Dsp^Flox/Flox^* mice at 14 weeks of age. A minimum of 36 tumors per group was graded. * p<0.01 by Fisher's exact test. (H) Same as G except IC lesions are separated into the IC1 and IC2 subclasses. * p<0.01 by the Chi-square test. (I) Tumors from *RT2+*; *Pdx1-Cre^ER^+*; *Dsp^WT/WT^*, *RT2+*; *Pdx1-Cre^ER^+*; *Dsp^Flox/WT^*, and *RT2+*; *Pdx1-Cre^ER^+*; *Dsp^Flox/Flox^* mice were genotyped for the presence of the *Cre* recombinase (∼530 bp), *β2 microglobulin* (∼290 bp), and the floxed (∼360 bp) or wild-type *Dsp* allele (∼230 bp). These same tumors were assessed for the recombination status of *Dsp*: wild-type allele (∼960 bp), non-recombined floxed allele (∼1200 bp), recombined floxed allele (∼650 bp). Scale bars represent 400 µm (A–C) and 200 µm (D–F).

While conditional genetic ablation of *Dsp* in the angiogenic islet dysplasias and incipient solid tumors of *RT2* mice had no discernible effects on tumor formation and subsequent tumor growth parameters, it did lead to an increase in tumor invasion. *RT2* mice develop a spectrum of tumor lesions, including non-invasive (IT), focally invasive (IC1), and broadly invasive (IC2) lesions (Figure 3A–F) [15]. Loss of *Dsp* resulted in a greater frequency of invasive tumors and a concomitant reduction in the percentage of non-invasive IT tumors in mice analyzed four weeks after genetic ablation of *Dsp* in incipient solid tumors (Figure 3G–H). Whereas ∼40% of total tumors could be classified as invasive carcinomas in control mice, greater than 60% of all tumors fell into this category in *RT2+*; *Pdx1-Cre^ER^+*; *Dsp^Flox/Flox^* mice (Figure 3G). Interestingly, this shift appears to result from selective progression to the focally invasive IC1 but not to the widely invasive IC2 tumors. Indeed, while there is no significant change in the development of IC2 lesions (approximately 10% of all tumors fall into this class regardless of *Dsp* status), more than 50% of tumors can be classified as IC1 lesions in *RT2+*; *Pdx1-Cre^ER^+*; *Dsp^Flox/Flox^* mice versus ∼30% in control mice (Figure 3H).

We confirmed that *Dsp* was in fact lost in these tumors by examining the recombination status of the *Dsp* allele by PCR. Tumors that were genotypically *Dsp^Flox/Flox^* showed near universal recombination of the *Dsp* allele, confirming that *Dsp* was lost in these tumors (Figure 3I). Tumors isolated from control *Dsp^WT/WT^* or *Dsp^Flox/WT^* mice showed no recombination or were heterozygous for the recombined and wild-type *Dsp* alleles respectively. Thus, we conclude that the conditional genetic ablation of *Dsp* in incipient tumors of *RT2* mice leads to increased local tumor invasion.

### *Cdh1* expression is maintained in IC1 tumor lesions regardless of *Dsp* status

**Figure 4:**
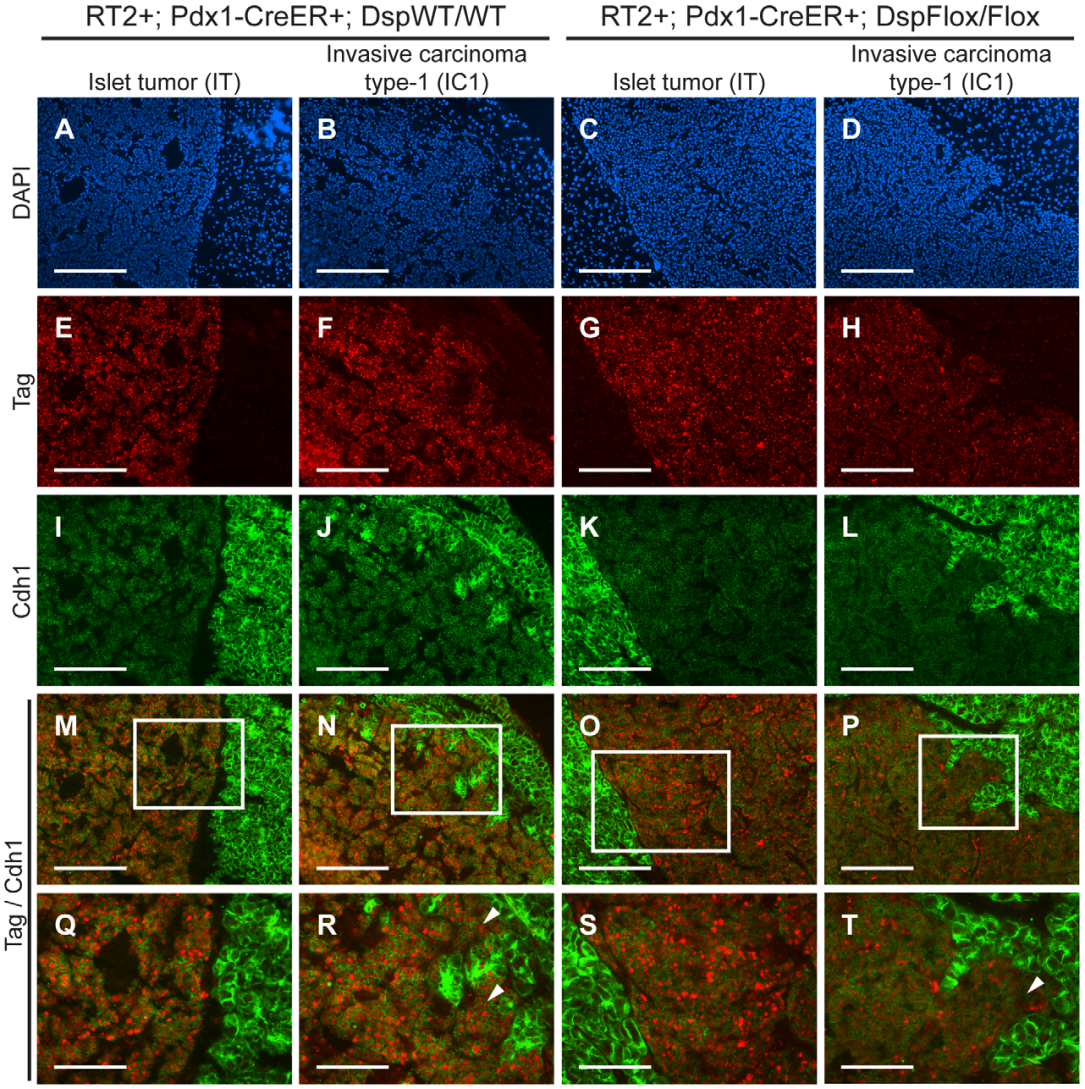
Genetic deletion of *desmoplakin* does not affect *cadherin 1* expression in *RT2* PNETs. *Cdh1* expression is maintained in the IC1 grade of tumors in both *RT2+*; *Pdx1-Cre^ER^+*; *Dsp^WT/WT^* and *RT2+*; *Pdx1-Cre^ER^+*; *Dsp^Flox/Flox^* mice. (A–D) Immunofluorescence staining with DAPI to reveal cellularity in IT and IC1 tumors in *RT2+*; *Pdx1-Cre^ER^+*; *Dsp^WT/WT^* and *RT2+*; *Pdx1-Cre^ER^+*; *Dsp^Flox/Flox^* mice. (E–H) Immunofluorescence staining for the oncoprotein T antigen (*Tag*). (I–L) Immunofluorescence staining for *Cdh1*. (M–P) Merge of *Tag* and *Cdh1* immunofluorescence staining (E–L). (Q–T) Higher magnification of the boxed regions in M–P. Arrowheads indicate regions of tumor invasion. Scale bars represent 200 µm (A–P) and 100 µm (Q–T).

We were intrigued that loss of *Dsp* led to an increase in the IC1 class but not in the IC2 class of invasive tumors. Since *Cdh1* also acts as a dominant invasion suppressor in this model, we examined its status in the tumors from *RT2+*; *Pdx1-Cre^ER^+*; *Dsp^Flox/Flox^* mice and littermate controls by immunohistochemistry. We found that *Cdh1* expression was maintained in the IT and IC1 tumors that developed regardless of *Dsp* status (Figure 4I–L). Tumor margins and regions of invasion were identified by staining for the *Tag* oncoprotein (Figure 4E–H). Indeed, *Cdh1* appeared to be expressed at comparable levels in IT and IC1 tumor lesions regardless of *Dsp* status (Figure 4M–T). Expression in IT and IC1 lesions of a second component of AJs, *junction plakoglobin* (*Jup*, also known as *gamma catenin*; MGI: 96650), was also unaffected by *Dsp* status (Figure S8), consistent with AJ function being maintained in these lesions despite the absence of *Dsp* and impaired/ablated desmosomal function. Lastly, *cadherin 2* (*Cdh2*, also known as *N-cadherin*; MGI: 88355), a marker of epithelial-mesenchymal transition (EMT), was expressed at readily detectable and comparable levels in IT and IC1 tumors regardless of *Dsp* status, as well as in the IC2 tumors that did not express *Cdh1* (Figure S9), consistent with the results of a previous study investigating determinants of progression to invasive carcinoma [8]; notably, there is no indication that activation of the invasive growth capability in this pathway involves an EMT, as reflected in differential expression of *Cdh2* or other markers of EMT. Given that the expression of both *Dsp* and *Cdh1* was lost in IC2 lesions, the most invasive class of *RT2* tumors, both in unmodified *RT2* mice and in tamoxifen-treated *RT2+*; *Pdx1-Cre^ER^+*; *Dsp^Flox/Flox^* mice (Figure 1 and data not shown), we infer that loss of *Dsp* by itself is sufficient to promote the development of focally invasive tumors while the additional loss of *Cdh1* is required to develop a more aggressive invasive tumor phenotype.

## Discussion

To date, much of the work on desmosomes in human disease has focused on their role in maintaining heart and skin integrity, where desmosomal defects are associated with cardiomyopathy and skin blistering conditions respectively [23]. More recently, a potential role for desmosomes in cancer progression has been suggested based on a variety of experimental clues [24]. For example, *in vitro* cell culture assays demonstrated that inhibiting desmosomal adhesion via blocking peptides caused morphological disorganization [25] while introduction of desmosomal components into a nonadhesive cell line resulted in increased cell aggregation and reduced cellular invasion *in vitro* [26]. These studies suggested that loss of desmosomal function might contribute to tumor invasion and malignancy, consistent with their role in maintaining cellular adhesion. (Our attempts to perform similar *in vitro* experiments using cell lines derived from *RT2* tumors [βTCs] were hindered by the fact that βTC cell lines express desmosomal components at low levels, presumably due to adaptations to culture, and generally perform poorly in migration/invasion assays – data not shown). In further support of the proposed role of desmosomes as a barrier to malignant progression, several pathology studies characterizing human cancers have shown that decreased or altered expression of desmosomal components, including *Dsp*, correlates with increased tumor invasion, advanced tumor grade, and poor patient prognosis, particularly in oral cancers where expression of desmosomal components are highly expressed in the normal oral mucosa [4], [5], [27]. Additionally, our bioinformatic analysis of human cancer databases confirmed that the expression of desmosomal genes is often decreased in a variety of human epithelial cancers as compared to normal tissues and is occasionally further decreased in more advanced grades of tumors (Table S3). The present study substantively extends this current state of knowledge by demonstrating that desmosomal adhesion can indeed act as a distinct barrier to the development of an invasive tumor phenotype in the *in vivo* setting of a genetically engineered mouse model of cancer.

We identified several components of desmosomes – *Dsp*, *Dsg2*, *desmocollin 2* (*Dsc2*; MGI: 103221), and *plakophilin 2* (*Pkp2*; MGI: 1914701) – whose expression was significantly downregulated in the highly invasive tumor lesions that develop in the *RT2* mouse model of PNET. These changes were reflected at the protein level as determined by immunostaining of non-invasive IT lesions and broadly invasive IC2 lesions. The simultaneous decrease in expression for multiple desmosomal genes suggests that there may be coordinated transcriptional regulation of desmosomal components. Prime candidates for such regulation include the transcription factors that regulate EMT, such as the *Snail* and *Twist* families of transcription factors [28]. Notably, however, we did not detect significant differential expression of such transcription factors in our microarray analysis comparing non-invasive IT and highly invasive IC2 PNETs (Dataset S1), and the expression of one prominent marker of EMT, *Cdh2*, was not obviously different between IT and IC2 lesions, consistent with the results of a previous study investigating determinants of the invasive phenotype using this same model of PNET [8]. Thus, the current evidence suggests that the acquisition of an invasive phenotype in this tumor type does not involve a classical EMT. Our results clearly demonstrate that the conditional genetic deletion of a single core desmosomal component, *Dsp*, promotes increased local tumor invasion in *RT2* mice, producing a phenocopy of such inferred transcriptional regulation in the normal circumstances of tumor progression.

While desmosomes play an integral role in maintaining epithelial integrity, they are by no means the only structure involved in cellular adhesion. In addition to desmosomes, several related structures, including AJs, contribute to maintaining cell-cell adhesion [7]. However, while desmosomes and AJs play related biological roles in terms of maintaining cellular adhesion and have similar structural compositions, it is worth noting that there are clear differences in the consequences of impaired desmosome adhesion versus impaired AJ adhesion on tumor phenotypes. An elegant functional genetic study demonstrated that *Cdh1*, a core member of AJs, acts as an invasion suppressor *in vivo*; targeting a transgene encoding a dominant-negative *Cdh1* molecule to the oncogene-expressing pancreatic β cells markedly accelerated tumor progression and led to significantly increased frequencies of invasive carcinomas and to the development of lymph node metastasis in this same mouse model of PNET [8]. In comparison, deletion of *Dsp* led to an increase in the frequency of the focally invasive IC1 grade of islet carcinomas but not the more widely aggressive IC2 carcinomas, and distant metastases were not observed (data not shown). One possible explanation for the differences in these phenotypic outcomes is the different roles that *Dsp* and *Cdh1* play within their respective adhesion complex. While *Cdh1* is a transmembrane protein that directly links cells together by forming homotypic interactions with other *Cdh1* molecules on neighboring cells [29], *Dsp* is an intracellular molecule that contributes to the overall stability of the desmosomal plaque and links this structure to the intermediate filaments [14]. Therefore, deletion of *Dsp* may attenuate but not totally abolish desmosomal function; if so, then the specific deletion of one of the desmosomal cadherins, *Dsc2* or *Dsg2*, might have a more pronounced effect on invasiveness. An additional explanation for the increase in the focally invasive IC1 fraction but not the broadly invasive IC2 fraction of invasive tumors following ablation of *Dsp* involves the observed maintenance of *Cdh1* and AJs. Expression of *Cdh1* as well as a second component of AJs, *Jup*, was retained in both the non-invasive IT tumors and in the now more prevalent focally invasive IC1 tumors following genetic deletion of *Dsp*. It would seem likely, in light of the aforementioned functional study in this same mouse model of cancer [8], that the preservation of *Cdh1* expression and of AJ function serves to maintain an additional, stronger brake on tumor invasion. Thus, while loss of *Dsp* and impairment of desmosomal adhesion leads to the focal invasion observed in IC1 lesions, the development of the broadly invasive phenotype found in IC2 lesions evidently requires the concomitant loss of *Cdh1*. Indeed, the IC2 tumor lesions that normally develop in *RT2* mice show a coordinated reduction in the expression of *Cdh1* and multiple desmosomal components (Table 1, Figure 1, and Figure S2). The apparently independent regulation of desmosomal and AJ adhesion is notable since AJ stability has been proposed to affect desmosomal stability and vice versa in other contexts [18], [30], [31], whereas *Cdh1* and *Jup* are evidently not affected by the deletion of *Dsp* during PNET tumorigenesis in *RT2* mice.

Interestingly, the genetic deletion of *Dsp* had no consequential effects on the other parameters of *RT2* tumorigenesis beyond invasion. Although it has been suggested that *Dsp* and other desmosomal components can affect cellular proliferation and apoptosis [32], [33], we did not observe any changes in tumor growth parameters following the genetic deletion of *Dsp* (Figure 2). Our results are consistent with one of the earliest studies to examine the role of *Dsp in vivo*, wherein a skin-specific deletion of *catenin alpha 1* (*Ctnna1*; MGI: 88274), the AJ homologue of *Dsp*, led to increased skin proliferation and hyperplasia whereas ablation of *Dsp* did not [34]. Thus, with regards to the *RT2* model of PNET and possibly other forms of cancer, it appears that desmosomes primarily serve to maintain cell-cell adhesion and hence suppress the acquisition of an invasive growth capability such that the observed downregulation of desmosomal genes results in the impairment of desmosomal function and a concomitant weakening in cellular adhesion without affecting other parameters of tumorigenesis.

**Figure 5:**
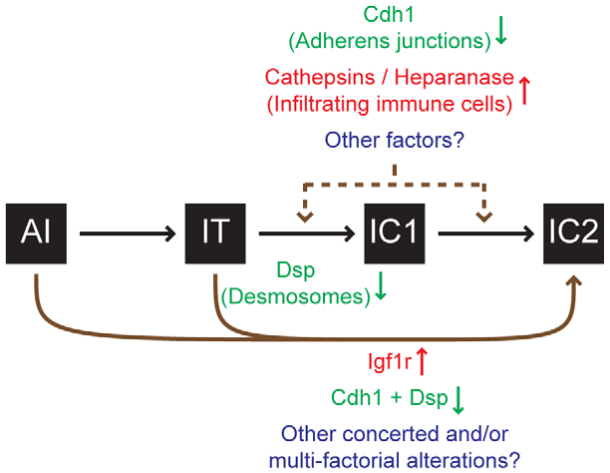
Progression to an invasive growth state is governed by multiple factors in the *RT2* model of PNET. Multiple factors impact the progression to an invasive phenotype as illustrated by the *RIP1-Tag2* (*RT2*) mouse model of pancreatic neuroendocrine tumorigenesis (PNET). This study demonstrates that the genetic deletion of *desmoplakin* (*Dsp*) and concomitant loss/attenuation of desmosomal adhesion can promote local tumor invasion, specifically to a focally invasive state typified by the IC1 tumor class. Other factors have also been demonstrated to affect tumor invasion in this model. Activation of *heparanase* or of *cathepsin* proteases [35]–[37] supplied by infiltrating immune cells or suppression of *cadherin 1* (*Cdh1*, also known as *E-cadherin*) [8] can each contribute to invasion. Upregulation of the *type-1 insulin-like growth factor receptor* (*Igf1r*) preferentially promotes progression to the IC2 stage, in part via a branched pathway from earlier neoplastic stages such as angiogenic islet dysplasias (AI), bypassing the canonical AI→IT→IC1→IC2 progression [15]. Future research may well identify additional factors that impact tumor invasion.

Finally, it is important to set these results into the broader context of knowledge about malignant progression to an invasive growth state in this stereotypical pathway of multistep tumorigenesis. While disrupted cell-cell adhesion caused by the reduced expression of *Cdh1* [8] and/or desmosomal genes (this report) clearly promotes invasive tumor growth, other factors are involved as well. Thus for example, increased expression of the *type-1 insulin-like growth factor receptor* (*Igf1r*; MGI: 96433) can drive these PNETs to acquire a highly invasive phenotype [15]. Additionally, the recruitment of immune cells to the margins of these PNETs has been shown to promote invasiveness, in part by supplying *cathepsin* proteases and *heparanase* (*Hpse*; MGI: 1343124) [35]–[37]. As such, multiple factors can impact the progression to invasiveness by varying degrees (Figure 5), and future research may well identify additional components. Irrespective, our results demonstrate that loss of desmosomal adhesion, as exemplified by the genetic deletion of *Dsp*, can enable a tumor to acquire an invasive phenotype. The functional study presented herein establishes desmosomal adhesion as a distinct and ostensibly independent suppressor of invasive tumor growth. This knowledge will likely contribute to a better understanding of the mechanisms governing tumor progression to an invasive growth state and may prove useful in evaluating invasive states of human cancers.

## Materials and Methods

### Ethics statement

All mice used in this study were housed and maintained in accordance with the University of California, San Francisco (UCSF) institutional guidelines governing the care of laboratory mice.

### Genetically engineered mice

The generation and characterization of the *RIP1-Tag2* (*RT2*) [10], *Dsp^Flox^* [18], and *Pdx1-Cre^ER^* [19] mouse lines have been previously reported. All mice were backcrossed a minimum of six generations into the *C57Bl/6* (*B6*) background (Charles River, Wilmington, MA) and then intercrossed to generate the specified genotypes. To induce *Cre^ER^* activity, mice were injected intraperitoneally with 100 µl of 10 mg/ml tamoxifen (Sigma, St. Louis, MO) suspended in peanut oil for five consecutive days beginning at 10 weeks of age. To relieve the effects of hypoglycemia induced by the insulin-secreting tumors, all *RT2* mice received 50% sugar food (Harlan Teklad, Madison, WI) beginning at 10 weeks of age.

### Tissue preparation, tumor analysis, and histology

Pancreata were isolated from 14-week-old mice and embedded in OCT (Sakura Finetek, Torrance, CA) on dry ice. Tumor number and tumor volume were quantified as previously described [12]. For histological analysis, frozen tissues were sectioned at 10 µm thickness, and every tenth section was stained with hematoxylin and eosin (Surgipath Medical Industries, Richmond, IL) using standard methods. Tumors were classified as a non-invasive islet tumor (IT), a focally invasive carcinoma type-1 (IC1), or a broadly invasive carcinoma type-2 (IC2) using a previously defined grading scheme [15].

### Laser capture microdissection and RNA purification and amplification

Fresh-frozen pancreatic sections (10 µm) from 14-week-old *RT2 B6* mice were fixed in cold 70% ethanol for 16 hours prior to laser capture microdissection (LCM). Sections were stained using a modified hematoxylin and eosin stain that preserves RNA integrity while allowing for the microscopic visualization of pancreatic structures [38]. LCM was performed using an Arcturus PixCell II laser capture microscope system (Molecular Devices, Sunnyvale, CA). Total RNA was isolated using the Arcturus PicoPure RNA Isolation kit (Molecular Devices, Sunnyvale, CA) and DNase I treated (Qiagen, Valencia, CA). Equal amounts of RNA (8 ng/lesion) from three independent IT or IC2 tumor lesions were pooled, and then cDNA was generated, amplified, and biotinylated using the Ovation Biotin System (NuGen, San Carlos, CA). Three independent pools per tumor class were generated for subsequent microarray analysis.

### Microarray analysis

Labeled cDNA was hybridized to Affymetrix Mouse Genome 430 2.0 arrays (Affymetrix, Santa Clara, CA) according to the manufacturer's specifications. Data were analyzed by the UCSF Helen Diller Family Comprehensive Cancer Center Biostatistics and Computational Biology Core. The data were normalized using a robust multi-chip averaging method utilizing the freely available R language. Linear models were fit for each pair of groups to be compared with log2 expression as the response and the tumor phenotype indicator as the independent variable using the limma package in Bioconductor. Moderated t-statistics were used, and p-values were adjusted by controlling the false discovery rate. A change in gene expression was identified as significant if the false discovery rate was less than 0.05, meaning that fewer than 5% of false findings would be expected among the genes declared to be differentially expressed.

### Immunohistochemical staining and analysis

Frozen tissues were sectioned at 10 µm thickness. For immunofluorescence staining, sections were fixed in cold acetone. For colorometric staining, sections were fixed in 10% Zn-buffered formalin (Medical Chemical Corporation, Torrance, CA), subjected to antigen retrieval using the Antigen Unmasking Solution (Vector Laboratories, Burlingame, CA), and blocked for endogenous peroxidase activity. Antibodies used in this study were as follows: rat anti-*cadherin 1* (Invitrogen, Carlsbad, CA); mouse anti-*desmoplakin I/II*, mouse anti-*desmoglein 1/2* (Fitzgerald, Concord, MA); mouse anti-*catenin beta 1*, mouse anti-*cadherin 2*, mouse anti-*junction plakoglobin* (BD Biosciences, San Jose, CA); guinea pig anti-*insulin* (Millipore, Billerica, MA); rabbit anti-T-antigen (Hanahan laboratory preparation); rabbit anti-Ki67 (Novus Biologicals, Littleton, CO); rhodamine red-X-conjugated donkey anti-mouse IgG, rhodamine red-X-conjugated donkey anti-rabbit IgG, FITC-conjugated donkey anti-rat IgG, FITC-conjugated donkey anti-guinea pig IgG, biotin-conjugated donkey anti-rabbit IgG (Jackson ImmunoResearch Laboratories, West Grove, PA). For mouse antibodies, non-specific binding was blocked using the Mouse on Mouse Blocking Reagent (Vector Laboratories, Burlingame, CA). Fluorescently labeled tissues were mounted with Vectashield mounting medium containing 4′,6-diamidino-2-phenylindole (DAPI) (Vector Laboratories, Burlingame, CA) to visualize cell nuclei. The TdT-mediated dUTP-digoxigenin nick-end labeling (TUNEL) assay was used to assess tumor apoptosis as previously described [15]. For colorometric staining, signal was amplified using the Vectastain Elite ABC kit (Vector Laboratories, Burlingame, CA), visualized using Nova Red substrate (Vector Laboratories, Burlingame, CA), and counterstained with hematoxylin. For Ki67 and TUNEL quantification, two to three random fields were obtained using a 40× objective lens from at least two tumors per mouse and at least five mice per group. The proliferation or apoptosis index was calculated as the percentage of total cells per field that were Ki67- or TUNEL-positive respectively using the MetaMorph software package (Molecular Devices, Sunnyvale, CA). For all other immunohistochemical analysis, two to three tumors per mouse from a minimum of five mice per indicated group were analyzed per staining condition. All images were captured using an Axio Imager bright field microscope or an Axio Scope fluorescence microscope and the AxioVision LE software package (Carl Zeiss, Thornwood, NY).

### Statistical analysis

Fisher's exact test and the chi-square test were used to compare tumor invasion metrics. The Mann-Whitney test was used to compare tumor burden, tumor number, tumor proliferation rates, tumor apoptosis rates, and body mass metrics. The Mann-Whitney and the Wilcoxon matched pairs test was used to compare fasting glucose metrics. For all statistical tests, a p-value of p≤0.05 was considered significant. All statistics were performed using the Prism software package (GraphPad Software, La Jolla, CA).

### Fasting glucose measurements

Animals were fasted overnight for 14–16 hours prior to the first tamoxifen injection and one week following the final tamoxifen injection. Fasting glucose levels were measured using a FreeStyle Freedom glucose meter (Abbott Laboratories, Abbott Park, IL).

### Tumor genotype analysis by polymerase chain reaction (PCR)

Tumor tissue was isolated directly from OCT embedded tissues, and genomic DNA was isolated using the QIAmp DNA Micro kit (Qiagen, Valencia, CA). PCR was performed using standard methods. Primers used were as follows: *Cre* (forward: 5′-CATGTTCAGGGATCGCCAGG-3′ and reverse: 5′-TGCGGTGCTAACCAGCGTTTT-3′); *β2 microglobulin* (forward: 5′-CACCGGAGAATGGGAAGCCGAA-3′ and reverse: 5′-TCCACACAGATGGAGCGTCCAG-3′); *Dsp*-WT/Flox (forward: 5′-GGTTGGGCCTCTCGAATCATGAGTGTCTAGCG-3′ and reverse: 5′-TGTCTGTTGCCATGTGATGCC-3′); *Dsp*-Recombined/Non-Recombined (forward: 5′-ACAGGCCAGATGAGATCACC-3′ and reverse: 5′-TGTCTGTTGCCATGTGATGCC-3′).

### Real-time quantitative PCR

Normal islets were isolated from six-week-old wild-type *B6* mice, and hyperplastic islets were isolated from six-week-old *RT2 B6* mice as previously described [39]. Angiogenic islets were isolated from nine-week-old *RT2 B6* mice by selection based on their red, hemorrhagic appearance following collagenase digestion of pancreata [39]. Islet tumors were excised from the surrounding exocrine pancreas from 14-week-old *RT2 B6* mice. Total RNA was purified using the RNeasy Mini kit (Qiagen, Valencia, CA) and DNase I treated (Qiagen, Valencia, CA). cDNA was synthesized using the iScript cDNA Synthesis kit (Bio-Rad Laboratories, Hercules, CA). Real-time quantitative PCR was performed using a 7900HT system (Applied Biosystems, Foster City, CA) (see Table S4 for a complete list of primers used in this study) according to the manufacturer's specifications.

## Supporting Information

Dataset S1 Microarray data for comparison of IT and IC2 classes of *RIP1-Tag2* tumors.
(8.87 MB XLS)

Figure S1 Expression of adherens junction and desmosomal components is decreased during PNET tumorigenesis in *RT2* mice and in human pancreatic neuroendocrine tumors. (A) Real-time quantitative PCR values for AJ components (*cadherin 1* [*Cdh1*], *catenin alpha 1* [*Ctnna1*], *catenin beta 1* [*Ctnnb1*], *catenin delta 1* [*Ctnnd1*], *junction plakoglobin* [*Jup*]) during the stages of *RT2* tumorigenesis - normal, hyperplastic, angiogenic, and tumor stage. Notably, in this analysis, whole ungraded *RT2* PNETs were analyzed without knowledge of their invasiveness in contrast to the analysis presented in Table 1, which involved microdissected tissue from either widely invasive IC2 tumors or from non-invasive IT tumors. (B) Same as A except for desmosomal components (*desmocollin 2* [*Dsc2*], *desmoglein 2* [*Dsg2*], *desmoplakin* [*Dsp*], *plakophilin 2* [*Pkp2*]). (C) Same as A except for the *insulin-like growth factor 2* (*Igf2*), a gene whose expression is known to increase at the mRNA level during the later stages of *RT2* tumorigenesis. (D) Real-time quantitative PCR values for *Cdh1*, *Dsg2*, and *Dsp* in pools of normal human pancreatic islets and individual human pancreatic neuroendocrine tumors (PNET). PNETs include ungraded primary and metastatic insulinomas, glucagonomas, and non-functional neuroendocrine tumors. Values are shown as the percent expression of the housekeeping genes *ribosomal protein L19* (*L19*) (A–C) or *glucuronidase beta* (*Gusb*) (D).
(1.22 MB TIF)

Figure S2 *Desmoglein 2* expression in *RT2* PNETs. Expression of *desmoglein 2* (*Dsg2*) is strongly reduced in the IC2 but not the IT grade of PNET in *RT2* mice. (A–C) H&E staining of a normal islet from a wild-type *B6* mouse and an IT and IC2 lesion from an end-stage *RT2* mouse. Dashed lines demarcate tumor margins. (D–F) Immunofluorescence staining with DAPI to reveal cellularity. (G–I) Immunofluorescence staining for *Cdh1*. (J–L) Immunofluorescence staining for *Dsg2*. (M–O) Merge of *Cdh1* and *Dsg2* immunofluorescence staining (G–L). (P–R) Higher magnification of the boxed regions in M–O. Scale bars represent 200 µm (A–O) and 100 µm (P–R).
(9.93 MB TIF)

Figure S3 *Catenin beta 1* expression in *RT2* PNETs. Expression of *catenin beta 1* (*Ctnnb1*) is maintained in both the IT and IC2 grades of PNET in *RT2* mice. (A–C) Immunofluorescence staining with DAPI to reveal cellularity of a normal islet from a wild-type *B6* mouse and an IT and IC2 tumor from an end-stage *RT2* mouse. (D–F) Immunofluorescence staining for *Cdh1*. (G–I) Immunofluorescence staining for *Ctnnb1*. (J–L) Merge of *Cdh1* and *Ctnnb1* immunofluorescence staining (D–I). (M–O) Higher magnification of the boxed regions in J–L. Scale bars represent 200 µm (A–L) and 100 µm (M–O).
(8.42 MB TIF)

Figure S4 Genetic deletion of *desmoplakin* does not affect *cadherin 1* expression in the pancreatic islets. Expression of *Cdh1* is maintained following conditional genetic deletion of *Dsp* in the pancreatic islets of control mice lacking the *RT2* oncogenic transgene. (A–C) H&E staining of pancreatic islets in *Pdx1-CreER+*; *DspWT/WT*, *Pdx1-CreER+*; *DspFlox/WT*, and *Pdx1-CreER+*; *DspFlox/Flox* mice at 14 weeks. *Cre* activity was induced at 10 weeks. (D–F) Immunofluorescence staining with DAPI to reveal cellularity. (G–I) Immunofluorescence staining for *Cdh1*. (J–L) Immunofluorescence staining for *Dsp*. (M–O) Merge of *Cdh1* and *Dsp* immunofluorescence staining (G–L). Scale bars represent 100 µm (A–O).
(8.40 MB TIF)

Figure S5 Genetic deletion of *desmoplakin* leads to decreased *desmoglein 2* expression but not *insulin* expression in the pancreatic islets. Expression of *Dsg2* but not *insulin* (*Ins*) is strongly reduced in the adult pancreatic islets following conditional genetic deletion of *Dsp* in mice lacking the *RT2* oncogenic transgene. (A–C) Immunofluorescence staining with DAPI to reveal cellularity in pancreatic islets in *Pdx1-CreER+*; *DspWT/WT*, *Pdx1-CreER+*; *DspFlox/WT*, and *Pdx1-CreER+*; *DspFlox/Flox* mice at 14 weeks. *Cre* activity was induced at 10 weeks. (D–F) Immunofluorescence staining for *Ins*. (G–I) Immunofluorescence staining for *Dsg2*. (J–L) Merge of *Ins* and *Dsg2* immunofluorescence staining (D–I). Scale bars represent 100 µm (A–L).
(6.84 MB TIF)

Figure S6 Genetic deletion of *desmoplakin* in the pancreatic islets does not affect multiple physiological parameters. Conditional genetic deletion of *Dsp* has no effect on the physiological parameters of body mass and islet function in regulating glucose levels. (A) Body mass of *Pdx1-CreER+; DspWT/WT*, *Pdx1-CreER+*; *DspFlox/WT*, *Pdx1-CreER+*; *DspFlox/Flox*, *RT2+*; *Pdx1-CreER+*; *DspWT/WT*, *RT2+*; *Pdx1-CreER+*; *DspFlox/WT*, and *RT2+*; *Pdx1-CreER+*; *DspFlox/Flox* mice at 14 weeks. *Cre* activity was induced at 10 weeks. Groups are not statistically different. (B) Fasting glucose levels in *Pdx1-CreER+*; *DspWT/WT*, *Pdx1-CreER+*; *DspFlox/WT*, and *Pdx1-CreER+*; *DspFlox/Flox* mice. *Cre* activity was induced at 10 weeks. Mice were fasted for 14–16 hours. Glucose levels were measured immediately prior to the first tamoxifen dose and one week following the last tamoxifen dose. Pre- and post-tamoxifen glucose levels within and between groups are not statistically different.
(10.19 MB TIF)

Figure S7 Tamoxifen does not affect the parameters of *RT2* tumorigenesis. Tamoxifen does not affect PNET tumorigenesis in unmodified *RT2* transgenic mice. Cohorts of male *RT2* mice that were *DspWT/WT* and that lacked the *Pdx1-CreER* allele were treated with five consecutive daily doses of tamoxifen or vehicle at 10 weeks of age and sacrificed 4 weeks later. (A–C) Tumor burden, tumor number, and body mass at time of sacrifice for *RT2* mice treated with tamoxifen or vehicle. Data shown are mean plus standard error. Groups are not statistically different for these metrics. (D) Quantification of tumor invasiveness represented as the percentage of IT lesions or total IC lesions (IC1+IC2) in *RT2* mice treated with tamoxifen or vehicle. A minimum of 76 tumors per group was graded. Groups are not statistically different. (E) Same as D except IC lesions are separated into the IC1 and IC2 subclasses. Groups are not statistically different.
(0.80 MB TIF)

Figure S8 Genetic deletion of *desmoplakin* does not affect *junction plakoglobin* expression in *RT2* PNETs. *Junction plakoglobin* (*Jup*, also known as *gamma catenin*) expression is maintained in the IC1 grade of tumors in both *RT2+*; *Pdx1-CreER+*; *DspWT/WT* and *RT2+*; *Pdx1-CreER+*; *DspFlox/Flox mice*. (A–D) Immunofluorescence staining for DAPI to reveal cellularity, *Cdh1*, *Jup*, and merge of *Cdh1* and *Jup* staining in an IT PNET from a *RT2+*; *Pdx1-CreER+*; *DspWT/WT* mouse. (E–H) Same as A–D except for an IC1 PNET from a *RT2+*; *Pdx1-CreER+*; *DspWT/WT* mouse. (I–L) Same as A–D except for an IT PNET from a *RT2+; Pdx1-CreER+*; *DspFlox/Flox* mouse. (M–P) Same as A–D except for an IC1 PNET from a *RT2+*; *Pdx1-CreER+*; *DspFlox/Flox* mouse. (Q–T) Same as A–D except for an IC2 PNET from an unmanipulated *RT2+* mouse. Arrowheads indicate regions of tumor invasion. Scale bars represent 200 µm.
(7.80 MB TIF)

Figure S9 Genetic deletion of *desmoplakin* does not affect *cadherin 2* expression in *RT2* PNETs. *Cadherin 2* (*Cdh2*, also known as *N-cadherin*) expression is maintained in the IC1 grade of tumors in both *RT2+*; *Pdx1-CreER+*; *DspWT/WT* and *RT2+*; *Pdx1-CreER+*; *DspFlox/Flox* mice. (A–D) Immunofluorescence staining for DAPI to reveal cellularity, *Cdh1*, *Cdh2*, and merge of *Cdh1* and *Cdh2* staining in an IT PNET from a *RT2+*; *Pdx1-CreER+*; *DspWT/WT* mouse. (E–H) Same as A–D except for an IC1 PNET from a *RT2+*; *Pdx1-CreER+*; *DspWT/WT* mouse. (I–L) Same as A-D except for an IT PNET from a *RT2+*; *Pdx1-CreER+*; *DspFlox/Flox* mouse. (M–P) Same as A–D except for an IC1 PNET from a *RT2+*; *Pdx1-CreER+*; *DspFlox/Flox* mouse. (Q–T) Same as A-D except for an IC2 PNET from an unmanipulated *RT2+* mouse. Arrowheads indicate regions of tumor invasion. Scale bars represent 200 µm.
(7.83 MB TIF)

Table S1 Genotyping of Pups Resulting from Intercross between *RIP1-Tag2+*; *DspFlox/WT* and *Pdx1-CreER+*; *DspFlox/WT* Mice.
(0.04 MB DOC)

Table S2 Gender Distribution of Pups Resulting from Intercross between *RIP1-Tag2+*; *DspFlox/WT* and *Pdx1-CreER+*; *DspFlox/WT* Mice.
(0.03 MB DOC)

Table S3 Bioinformatic Assessment of Desmosomal Gene Expression in Human Cancers.
(0.06 MB DOC)

Table S4 List of primers used for quantitative PCR.
(0.04 MB DOC)
