# Supplementary figures and images for: Genetic Deletion of the Desmosomal Component Desmoplakin Promotes Tumor Microinvasion in a Mouse Model of Pancreatic Neuroendocrine Carcinogenesis
Source: PLoS Genet. 2010 Sep 16;6(9):e1001120. doi: 10.1371/journal.pgen.1001120 (PMC2940733; doi:10.1371/journal.pgen.1001120)

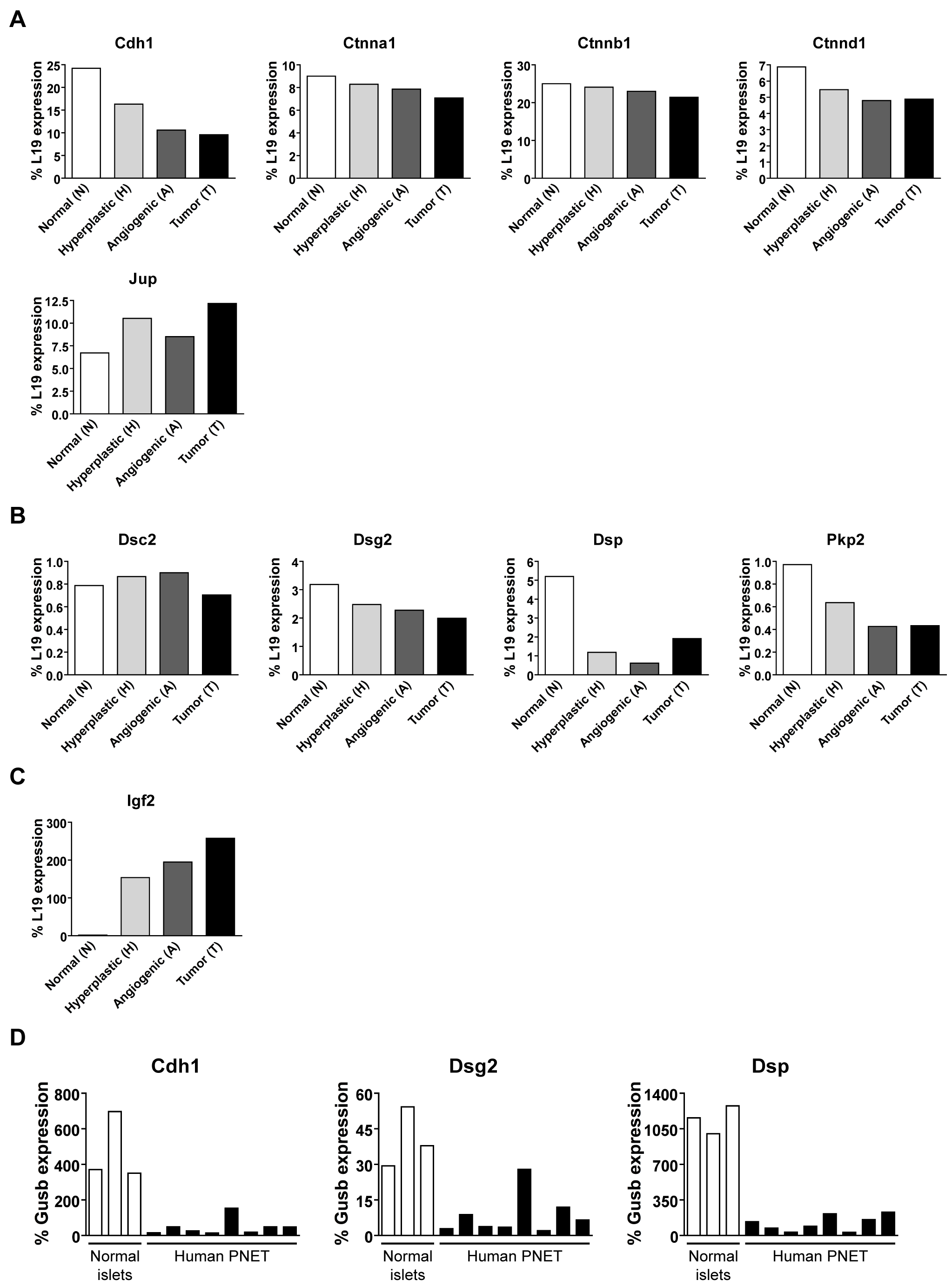

Supplement: Figure S1 — Expression of adherens junction and desmosomal components is decreased during PNET tumorigenesis in RT2 mice and in human pancreatic neuroendocrine tumors. (A) Real-time quantitative PCR values for AJ components (cadherin 1 [Cdh1], catenin alpha 1 [Ctnna1], catenin beta 1 [Ctnnb1], catenin delta 1 [Ctnnd1], junction plakoglobin [Jup]) during the stages of RT2 tumorigenesis - normal, hyperplastic, angiogenic, and tumor stage. Notably, in this analysis, whole ungraded RT2 PNETs were analyzed without knowledge of their invasiveness in contrast to the analysis presented in Table 1, which involved microdissected tissue from either widely invasive IC2 tumors or from non-invasive IT tumors. (B) Same as A except for desmosomal components (desmocollin 2 [Dsc2], desmoglein 2 [Dsg2], desmoplakin [Dsp], plakophilin 2 [Pkp2]). (C) Same as A except for the insulin-like growth factor 2 (Igf2), a gene whose expression is known to increase at the mRNA level during the later stages of RT2 tumorigenesis. (D) Real-time quantitative PCR values for Cdh1, Dsg2, and Dsp in pools of normal human pancreatic islets and individual human pancreatic neuroendocrine tumors (PNET). PNETs include ungraded primary and metastatic insulinomas, glucagonomas, and non-functional neuroendocrine tumors. Values are shown as the percent expression of the housekeeping genes ribosomal protein L19 (L19) (A–C) or glucuronidase beta (Gusb) (D). (1.22 MB TIF) [file pgen.1001120.s002.tif]

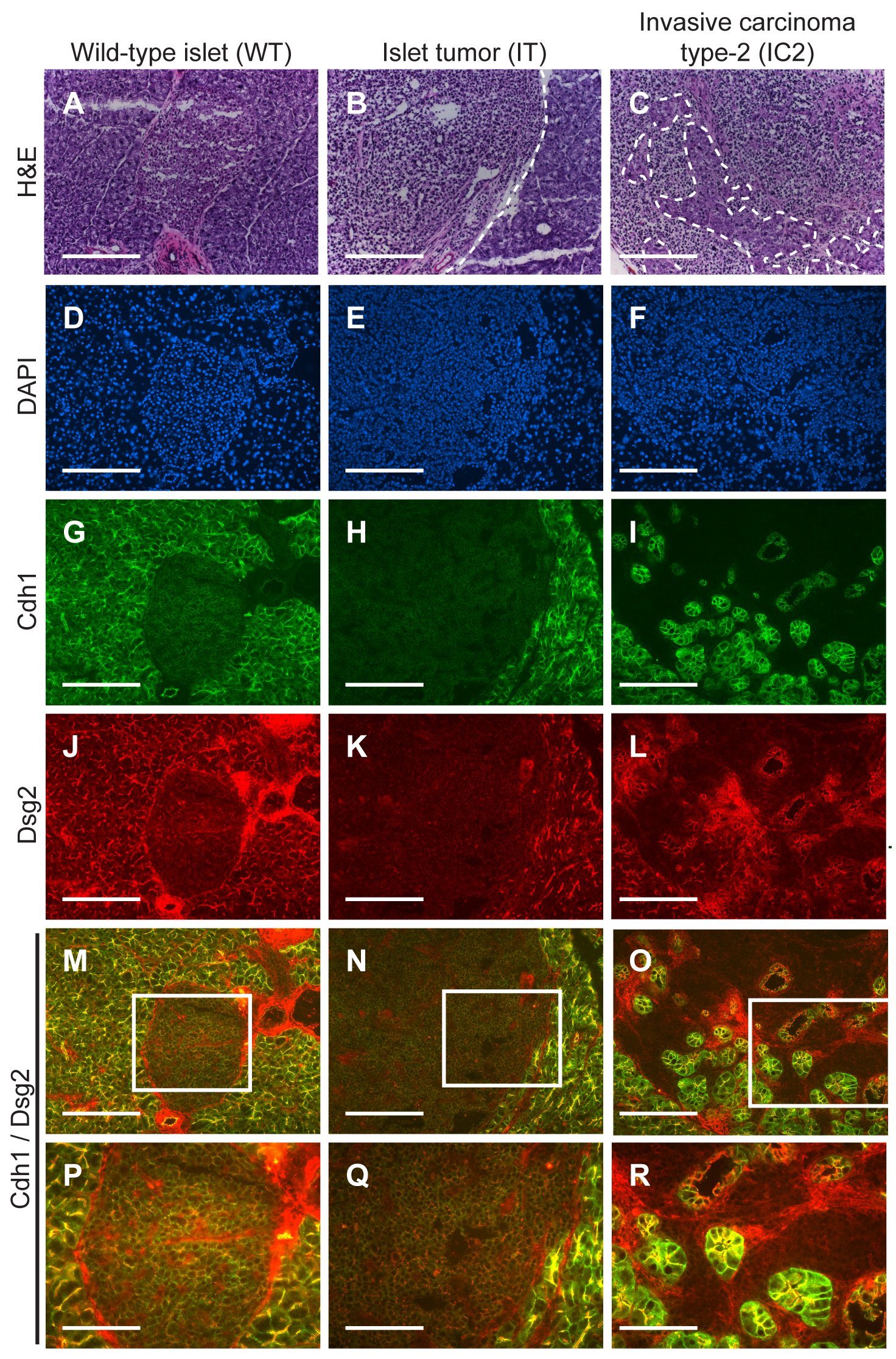

Supplement: Figure S2 — Desmoglein 2 expression in RT2 PNETs. Expression of desmoglein 2 (Dsg2) is strongly reduced in the IC2 but not the IT grade of PNET in RT2 mice. (A–C) H&E staining of a normal islet from a wild-type B6 mouse and an IT and IC2 lesion from an end-stage RT2 mouse. Dashed lines demarcate tumor margins. (D–F) Immunofluorescence staining with DAPI to reveal cellularity. (G–I) Immunofluorescence staining for Cdh1. (J–L) Immunofluorescence staining for Dsg2. (M–O) Merge of Cdh1 and Dsg2 immunofluorescence staining (G–L). (P–R) Higher magnification of the boxed regions in M–O. Scale bars represent 200 µm (A–O) and 100 µm (P–R). (9.93 MB TIF) [file pgen.1001120.s003.tif]

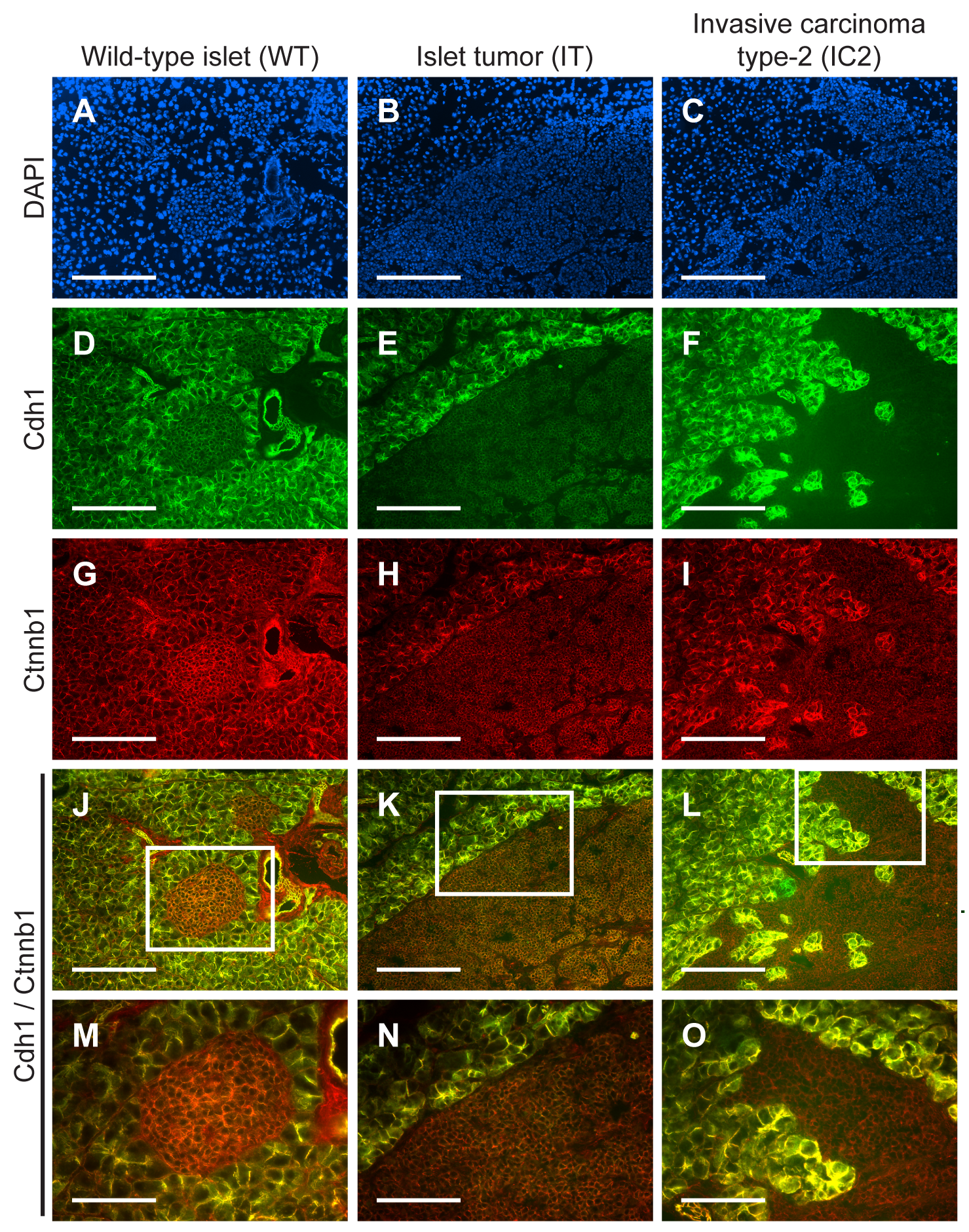

Supplement: Figure S3 — Catenin beta 1 expression in RT2 PNETs. Expression of catenin beta 1 (Ctnnb1) is maintained in both the IT and IC2 grades of PNET in RT2 mice. (A–C) Immunofluorescence staining with DAPI to reveal cellularity of a normal islet from a wild-type B6 mouse and an IT and IC2 tumor from an end-stage RT2 mouse. (D–F) Immunofluorescence staining for Cdh1. (G–I) Immunofluorescence staining for Ctnnb1. (J–L) Merge of Cdh1 and Ctnnb1 immunofluorescence staining (D–I). (M–O) Higher magnification of the boxed regions in J–L. Scale bars represent 200 µm (A–L) and 100 µm (M–O). (8.42 MB TIF) [file pgen.1001120.s004.tif]

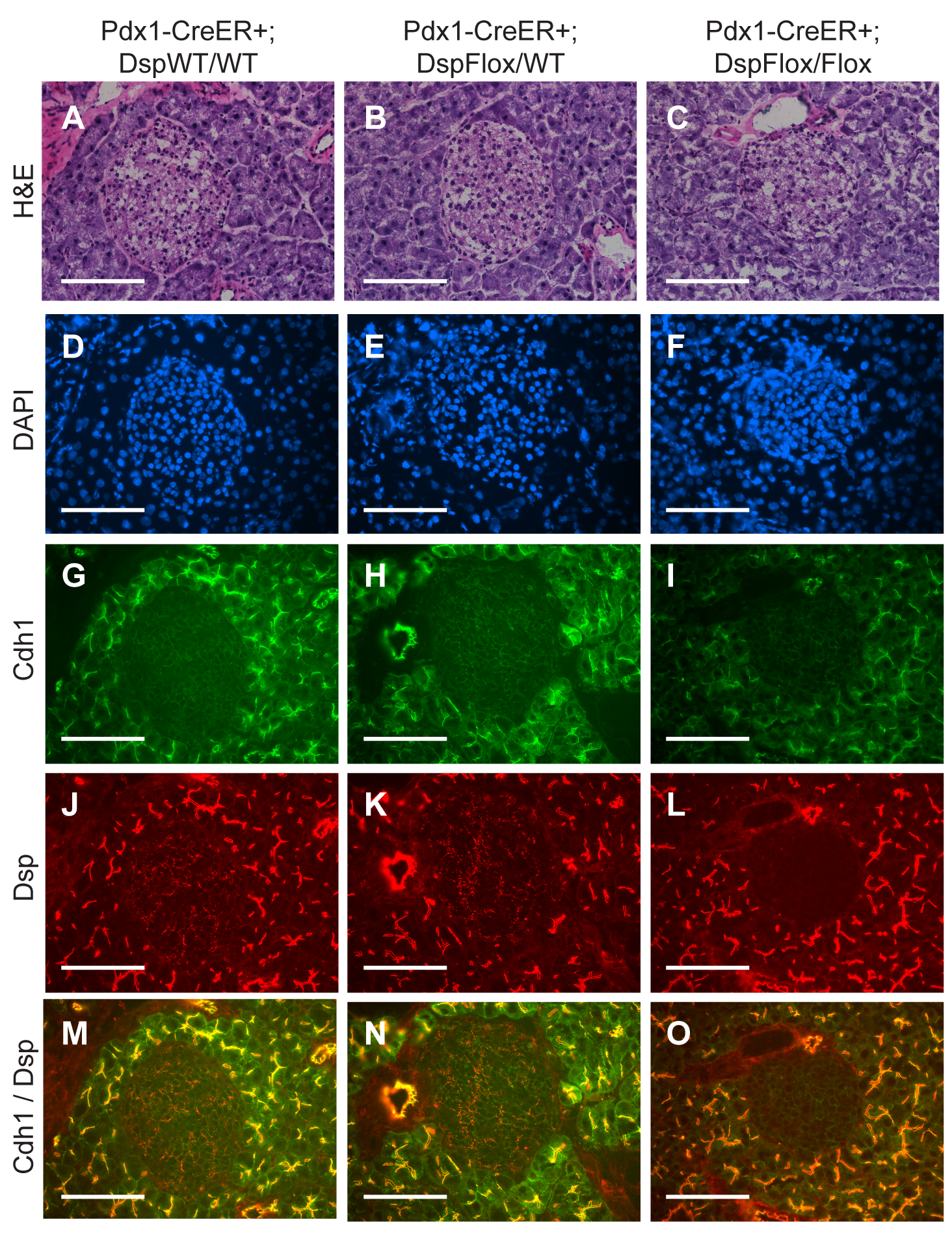

Supplement: Figure S4 — Genetic deletion of desmoplakin does not affect cadherin 1 expression in the pancreatic islets. Expression of Cdh1 is maintained following conditional genetic deletion of Dsp in the pancreatic islets of control mice lacking the RT2 oncogenic transgene. (A–C) H&E staining of pancreatic islets in Pdx1-CreER+; DspWT/WT, Pdx1-CreER+; DspFlox/WT, and Pdx1-CreER+; DspFlox/Flox mice at 14 weeks. Cre activity was induced at 10 weeks. (D–F) Immunofluorescence staining with DAPI to reveal cellularity. (G–I) Immunofluorescence staining for Cdh1. (J–L) Immunofluorescence staining for Dsp. (M–O) Merge of Cdh1 and Dsp immunofluorescence staining (G–L). Scale bars represent 100 µm (A–O). (8.40 MB TIF) [file pgen.1001120.s005.tif]

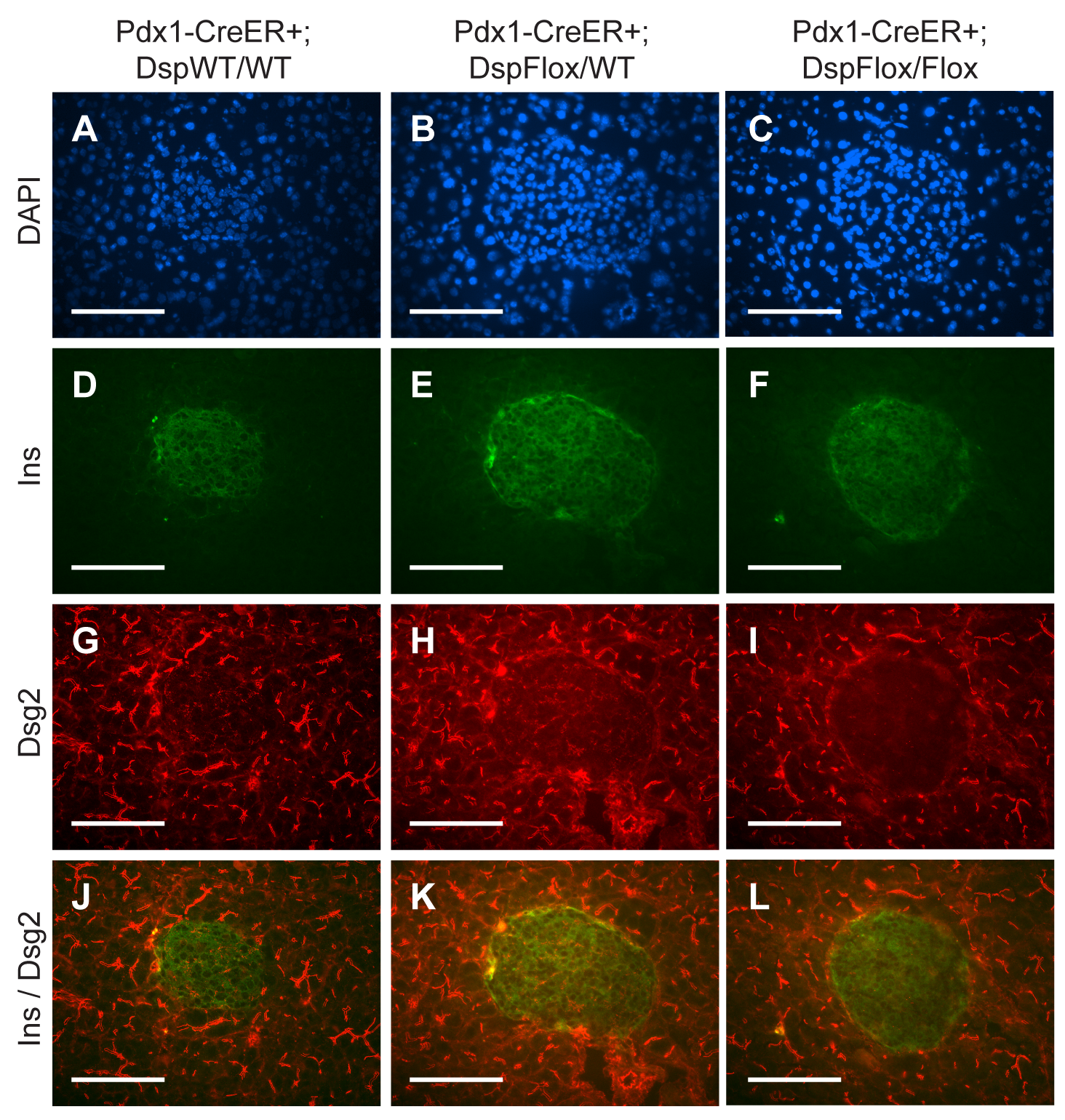

Supplement: Figure S5 — Genetic deletion of desmoplakin leads to decreased desmoglein 2 expression but not insulin expression in the pancreatic islets. Expression of Dsg2 but not insulin (Ins) is strongly reduced in the adult pancreatic islets following conditional genetic deletion of Dsp in mice lacking the RT2 oncogenic transgene. (A–C) Immunofluorescence staining with DAPI to reveal cellularity in pancreatic islets in Pdx1-CreER+; DspWT/WT, Pdx1-CreER+; DspFlox/WT, and Pdx1-CreER+; DspFlox/Flox mice at 14 weeks. Cre activity was induced at 10 weeks. (D–F) Immunofluorescence staining for Ins. (G–I) Immunofluorescence staining for Dsg2. (J–L) Merge of Ins and Dsg2 immunofluorescence staining (D–I). Scale bars represent 100 µm (A–L). (6.84 MB TIF) [file pgen.1001120.s006.tif]

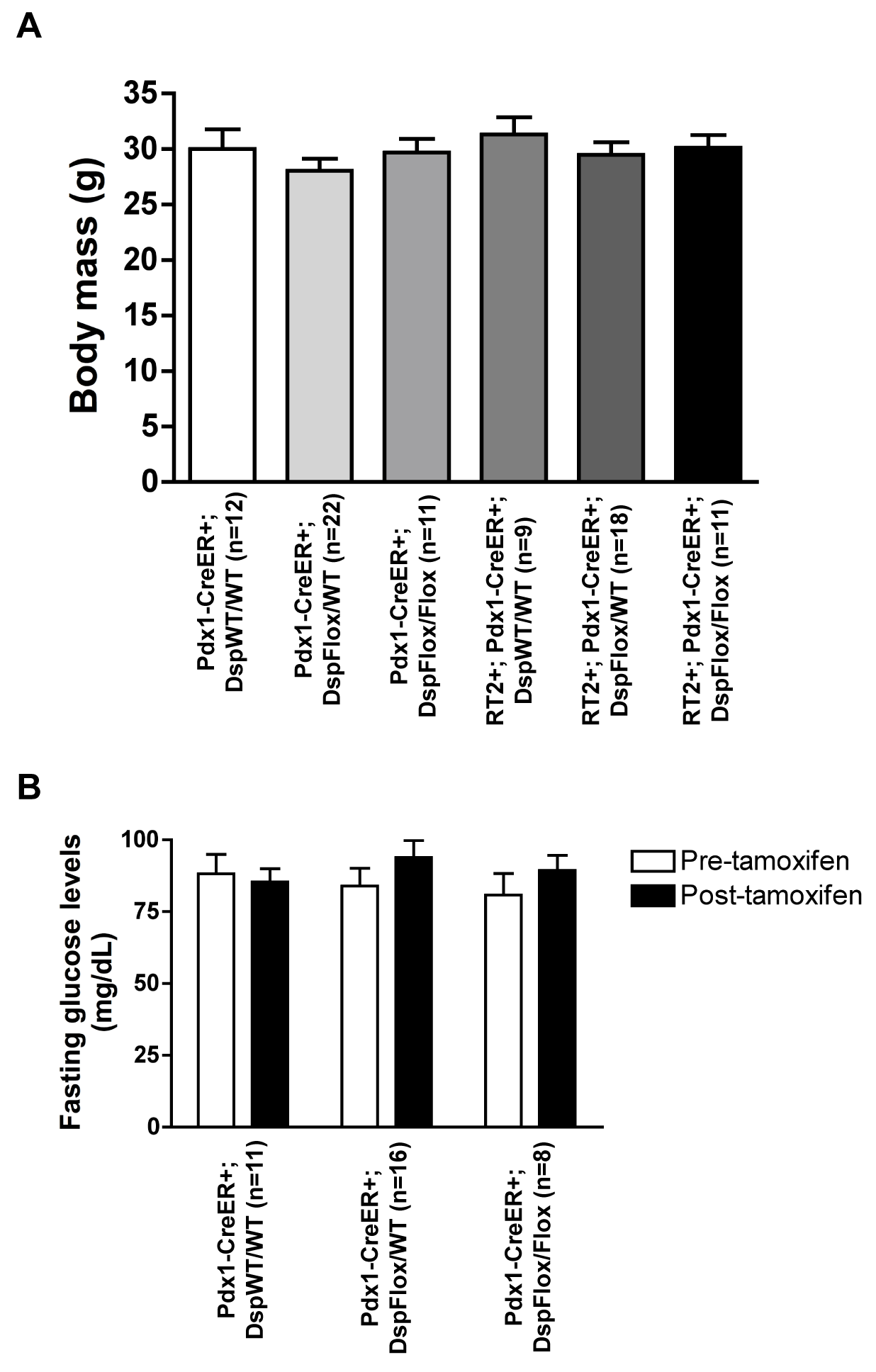

Supplement: Figure S6 — Genetic deletion of desmoplakin in the pancreatic islets does not affect multiple physiological parameters. Conditional genetic deletion of Dsp has no effect on the physiological parameters of body mass and islet function in regulating glucose levels. (A) Body mass of Pdx1-CreER+; DspWT/WT, Pdx1-CreER+; DspFlox/WT, Pdx1-CreER+; DspFlox/Flox, RT2+; Pdx1-CreER+; DspWT/WT, RT2+; Pdx1-CreER+; DspFlox/WT, and RT2+; Pdx1-CreER+; DspFlox/Flox mice at 14 weeks. Cre activity was induced at 10 weeks. Groups are not statistically different. (B) Fasting glucose levels in Pdx1-CreER+; DspWT/WT, Pdx1-CreER+; DspFlox/WT, and Pdx1-CreER+; DspFlox/Flox mice. Cre activity was induced at 10 weeks. Mice were fasted for 14–16 hours. Glucose levels were measured immediately prior to the first tamoxifen dose and one week following the last tamoxifen dose. Pre- and post-tamoxifen glucose levels within and between groups are not statistically different. (10.19 MB TIF) [file pgen.1001120.s007.tif]

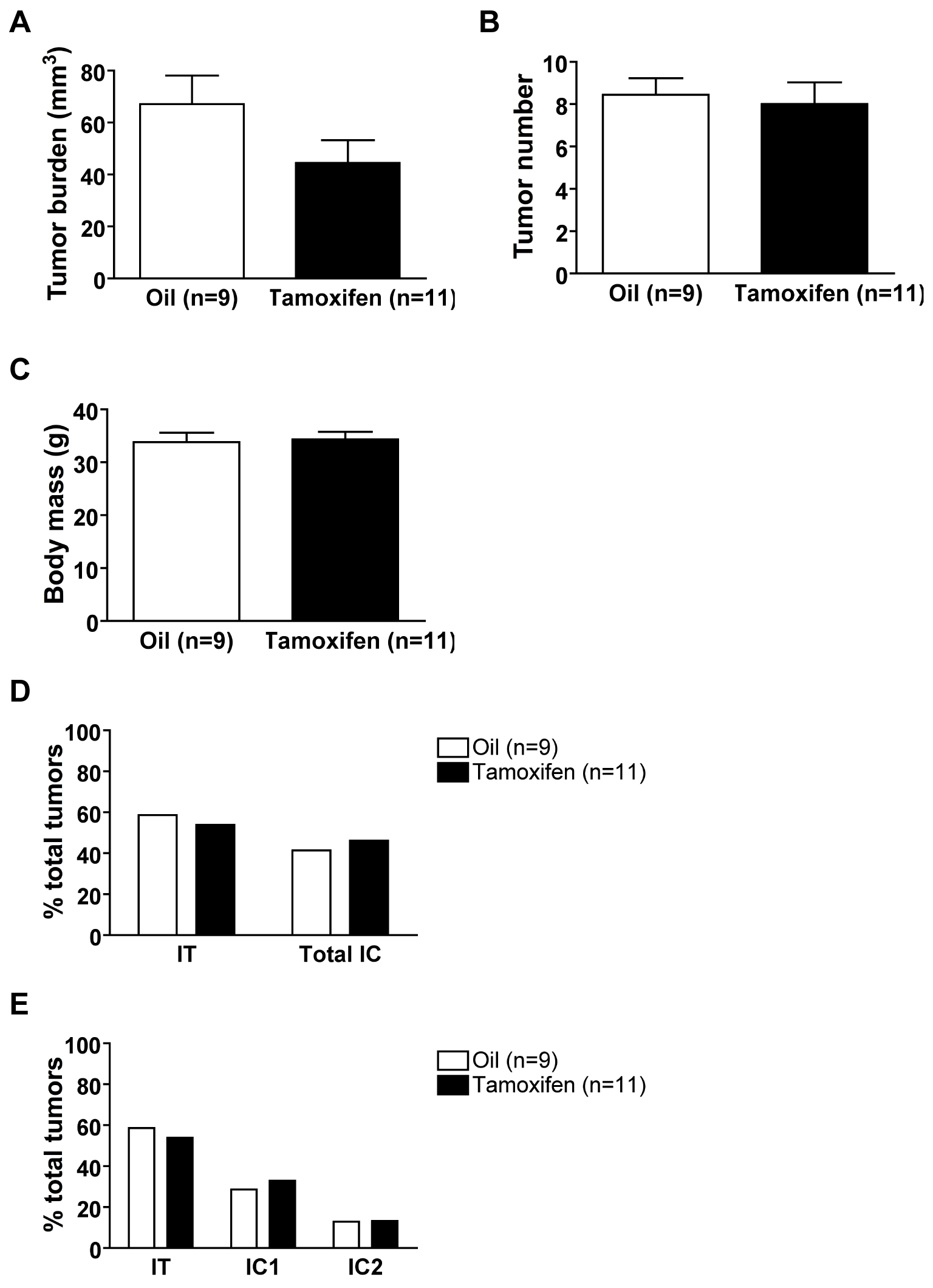

Supplement: Figure S7 — Tamoxifen does not affect the parameters of RT2 tumorigenesis. Tamoxifen does not affect PNET tumorigenesis in unmodified RT2 transgenic mice. Cohorts of male RT2 mice that were DspWT/WT and that lacked the Pdx1-CreER allele were treated with five consecutive daily doses of tamoxifen or vehicle at 10 weeks of age and sacrificed 4 weeks later. (A–C) Tumor burden, tumor number, and body mass at time of sacrifice for RT2 mice treated with tamoxifen or vehicle. Data shown are mean plus standard error. Groups are not statistically different for these metrics. (D) Quantification of tumor invasiveness represented as the percentage of IT lesions or total IC lesions (IC1+IC2) in RT2 mice treated with tamoxifen or vehicle. A minimum of 76 tumors per group was graded. Groups are not statistically different. (E) Same as D except IC lesions are separated into the IC1 and IC2 subclasses. Groups are not statistically different. (0.80 MB TIF) [file pgen.1001120.s008.tif]

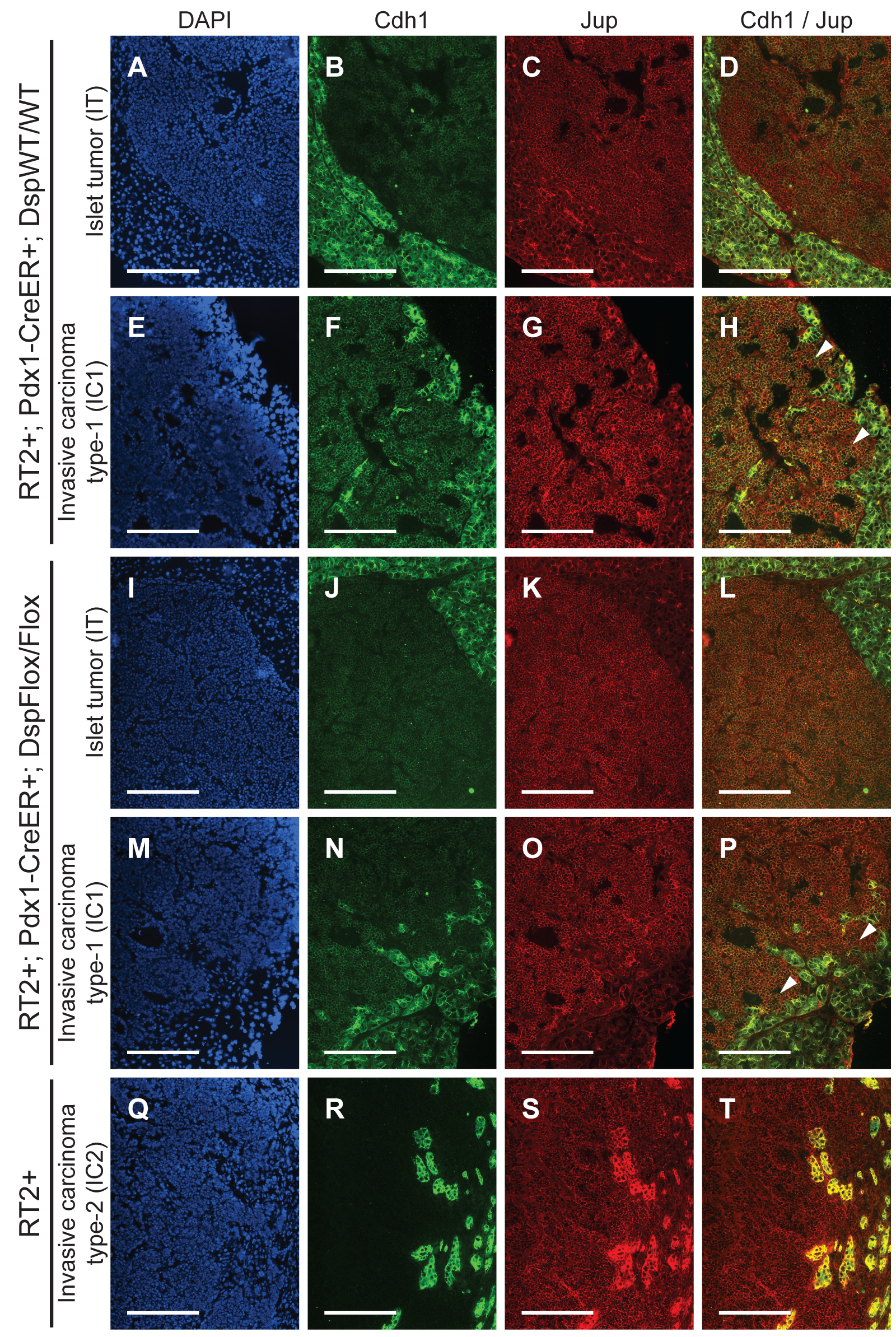

Supplement: Figure S8 — Genetic deletion of desmoplakin does not affect junction plakoglobin expression in RT2 PNETs. Junction plakoglobin (Jup, also known as gamma catenin) expression is maintained in the IC1 grade of tumors in both RT2+; Pdx1-CreER+; DspWT/WT and RT2+; Pdx1-CreER+; DspFlox/Flox mice. (A–D) Immunofluorescence staining for DAPI to reveal cellularity, Cdh1, Jup, and merge of Cdh1 and Jup staining in an IT PNET from a RT2+; Pdx1-CreER+; DspWT/WT mouse. (E–H) Same as A–D except for an IC1 PNET from a RT2+; Pdx1-CreER+; DspWT/WT mouse. (I–L) Same as A–D except for an IT PNET from a RT2+; Pdx1-CreER+; DspFlox/Flox mouse. (M–P) Same as A–D except for an IC1 PNET from a RT2+; Pdx1-CreER+; DspFlox/Flox mouse. (Q–T) Same as A–D except for an IC2 PNET from an unmanipulated RT2+ mouse. Arrowheads indicate regions of tumor invasion. Scale bars represent 200 µm. (7.80 MB TIF) [file pgen.1001120.s009.tif]

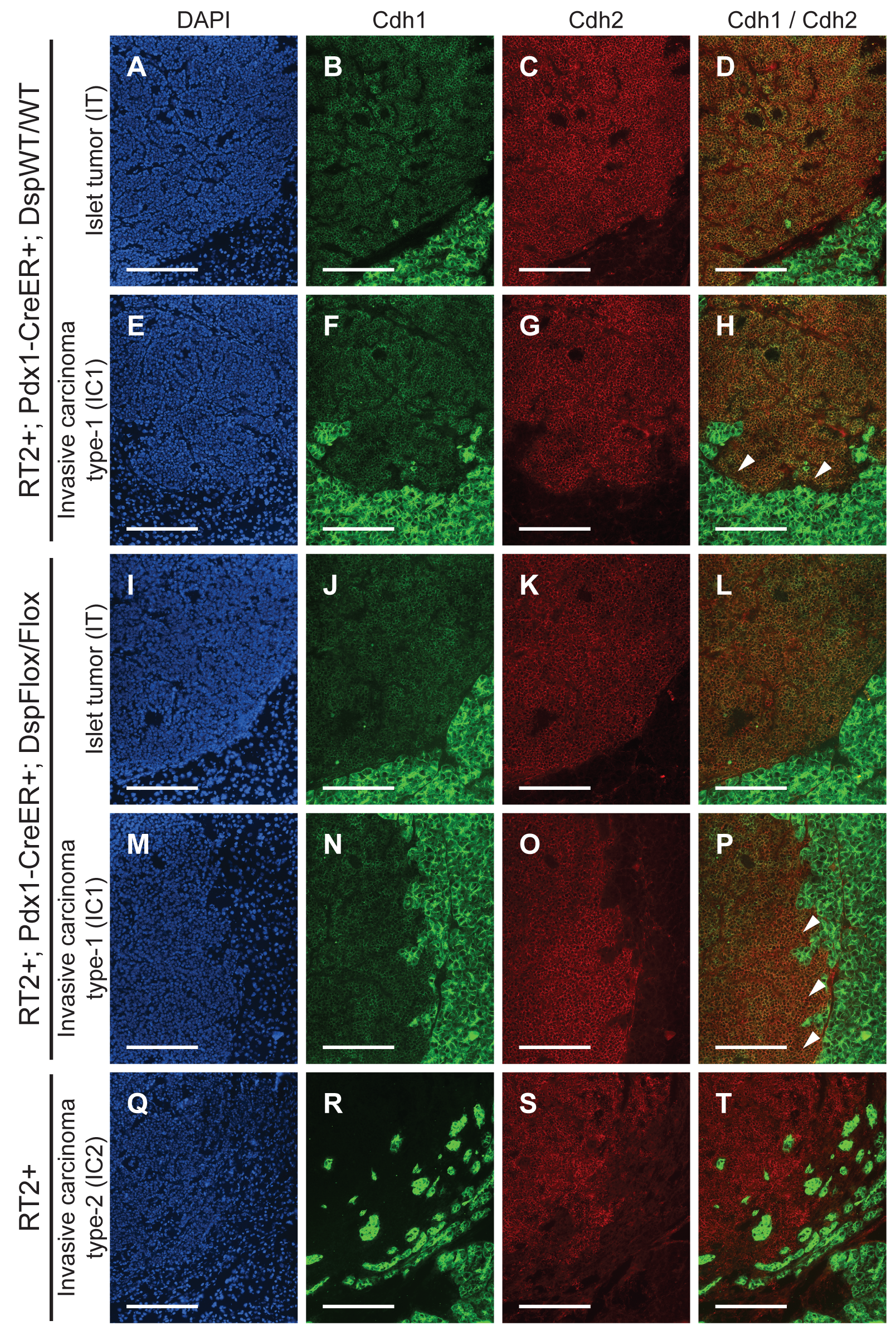

Supplement: Figure S9 — Genetic deletion of desmoplakin does not affect cadherin 2 expression in RT2 PNETs. Cadherin 2 (Cdh2, also known as N-cadherin) expression is maintained in the IC1 grade of tumors in both RT2+; Pdx1-CreER+; DspWT/WT and RT2+; Pdx1-CreER+; DspFlox/Flox mice. (A–D) Immunofluorescence staining for DAPI to reveal cellularity, Cdh1, Cdh2, and merge of Cdh1 and Cdh2 staining in an IT PNET from a RT2+; Pdx1-CreER+; DspWT/WT mouse. (E–H) Same as A–D except for an IC1 PNET from a RT2+; Pdx1-CreER+; DspWT/WT mouse. (I–L) Same as A-D except for an IT PNET from a RT2+; Pdx1-CreER+; DspFlox/Flox mouse. (M–P) Same as A–D except for an IC1 PNET from a RT2+; Pdx1-CreER+; DspFlox/Flox mouse. (Q–T) Same as A-D except for an IC2 PNET from an unmanipulated RT2+ mouse. Arrowheads indicate regions of tumor invasion. Scale bars represent 200 µm. (7.83 MB TIF) [file pgen.1001120.s010.tif]
