# Supplementary material for: Genetic Deletion of the Desmosomal Component Desmoplakin Promotes Tumor Microinvasion in a Mouse Model of Pancreatic Neuroendocrine Carcinogenesis
Source: PLoS Genet. 2010 Sep 16;6(9):e1001120. doi: 10.1371/journal.pgen.1001120 (PMC2940733; doi:10.1371/journal.pgen.1001120)
Supplement: Table S1 — Genotyping of Pups Resulting from Intercross between RIP1-Tag2+; DspFlox/WT and Pdx1-CreER+; DspFlox/WT Mice. (0.04 MB DOC) [file pgen.1001120.s011.doc]

**Supplemental Table 1. Genotyping of Pups Resulting from Intercross between *RIP1-Tag2+; Dsp^Flox/WT^*and *Pdx1-CreER+; Dsp^Flox/WT^* Mice.**

| **Genotype** | **Expected** | **Observed** |
| --- | --- | --- |
| *RIP1-Tag2-; Pdx1-CreER-; Dsp^WT/WT^* | 6.25% | 6.9% (14) |
| *RIP1-Tag2-; Pdx1-CreER-; Dsp^Flox/WT^* | 12.5% | 17.2% (35) |
| *RIP1-Tag2-; Pdx1-CreER-; Dsp^Flox/Flox^* | 6.25% | 6.4% (13) |
| *RIP1-Tag2+; Pdx1-CreER-; Dsp^WT/WT^* | 6.25% | 7.4% (15) |
| *RIP1-Tag2+; Pdx1-CreER-; Dsp^Flox/WT^* | 12.5% | 12.8% (26) |
| *RIP1-Tag2+; Pdx1-CreER-; Dsp^Flox/Flox^* | 6.25% | 6.4% (13) |
| *RIP1-Tag2-; Pdx1-CreER+; Dsp^WT/WT^* | 6.25% | 6.4% (13) |
| *RIP1-Tag2-; Pdx1-CreER+; Dsp^Flox/WT^* | 12.5% | 10.8% (22) |
| *RIP1-Tag2-; Pdx1-CreER+; Dsp^Flox/Flox^* | 6.25% | 5.9% (12) |
| *RIP1-Tag2+; Pdx1-CreER+; Dsp^WT/WT^* | 6.25% | 4.9% (10) |
| *RIP1-Tag2+; Pdx1-CreER+; Dsp^Flox/WT^* | 12.5% | 9.4% (19) |
| *RIP1-Tag2+; Pdx1-CreER+; Dsp^Flox/Flox^* | 6.25% | 5.4% (11) |

The expected and observed frequency of each genotype is shown as a percentage, with the absolute numbers of individuals shown in parentheses.
