# Supplementary material for: Genetic Deletion of the Desmosomal Component Desmoplakin Promotes Tumor Microinvasion in a Mouse Model of Pancreatic Neuroendocrine Carcinogenesis
Source: PLoS Genet. 2010 Sep 16;6(9):e1001120. doi: 10.1371/journal.pgen.1001120 (PMC2940733; doi:10.1371/journal.pgen.1001120)
Supplement: Table S2 — Gender Distribution of Pups Resulting from Intercross between RIP1-Tag2+; DspFlox/WT and Pdx1-CreER+; DspFlox/WT Mice. (0.03 MB DOC) [file pgen.1001120.s012.doc]

**Supplemental Table 2. Gender Distribution of Pups Resulting from Intercross between *RIP1-Tag2+; Dsp^Flox/WT^*and *Pdx1-CreER+; Dsp^Flox/WT^* Mice.**

| **Gender** | **Expected** | **Observed** |
| --- | --- | --- |
| Female | 50% | 48.8% (99) |
| Male | 50% | 51.2% (104) |

The expected and observed frequency of each genotype is shown as a percentage, with the absolute numbers of individuals shown in parentheses.
