# Supplementary material for: Genetic Deletion of the Desmosomal Component Desmoplakin Promotes Tumor Microinvasion in a Mouse Model of Pancreatic Neuroendocrine Carcinogenesis
Source: PLoS Genet. 2010 Sep 16;6(9):e1001120. doi: 10.1371/journal.pgen.1001120 (PMC2940733; doi:10.1371/journal.pgen.1001120)
Supplement: Table S3 — Bioinformatic Assessment of Desmosomal Gene Expression in Human Cancers. (0.06 MB DOC) [file pgen.1001120.s013.doc]

**Supplemental Table 3. Bioinformatic Assessment of Desmosomal Gene Expression in Human Cancers.**

| **Gene** | **Class 1^a^** | **Class 2^a^** | **Fold-change^b^** | **P-value** | **Oncomine category** | **Platform** | **Source** | **Citation** |
| --- | --- | --- | --- | --- | --- | --- | --- | --- |
| *Dsc2* | Fetal kidney (3) | Clear cell sarcoma of the kidney (14) | -2.353 | 6.77E-04 | Cancer versus normal | Human genome U133A array | Cutliffe renal | Clinical Cancer Research 2005/11/15 |
| *Dsc2* | Skin (7) | Cutaneous melanoma (45) | -1.911 | 4.00E-03 | Cancer versus normal | Human genome U133A array | Talantov melanoma | Clinical Cancer Research 2005/10/15 |
| *Dsc2* | Breast (3) | Lobular breast carcinoma (21) | -2.207 | 8.93E-05 | Cancer versus normal | Undefined on Oncomine | Zhao breast | Molecular Biology of the Cell 2004/06/01 |
| *Dsg2* | Fetal kidney (3) | Clear cell sarcoma of the kidney (14) | -16.174 | 3.36E-06 | Cancer versus normal | Human genome U133A array | Cutliffe renal | Clinical Cancer Research 2005/11/15 |
| *Dsg2* | Brain (23) | Oligodendroglioma (50) | -1.519 | 8.00E-03 | Cancer versus normal | Human genome U133 Plus 2.0 array | Sun brain | Cancer Cell 2006/04/01 |
| *Dsg2* | Skin (7) | Cutaneous melanoma (45) | -11.919 | 8.82E-07 | Cancer versus normal | Human genome U133A array | Talantov melanoma | Clinical Cancer Research 2005/10/15 |
| *Dsp* | Fetal kidney (3) | Clear cell sarcoma of the kidney (14) | -17.869 | 1.82E-08 | Cancer versus normal | Human genome U133A array | Cutliffe renal | Clinical Cancer Research 2005/11/15 |
| *Dsp* | Brain (23) | Glioblastoma (81) | -1.820 | 1.79E-05 | Cancer versus normal | Human genome U133 Plus 2.0 array | Sun brain | Cancer Cell 2006/04/01 |
| *Dsp* | Brain (23) | Oligodendroglioma (50) | -1.824 | 1.41E-05 | Cancer versus normal | Human genome U133 Plus 2.0 array | Sun brain | Cancer Cell 2006/04/01 |
| *Dsp* | Skin (7) | Cutaneous melanoma (45) | -66.700 | 6.06E-18 | Cancer versus normal | Human genome U133A array | Talantov melanoma | Clinical Cancer Research 2005/10/15 |
| *Dsp* | Breast (3) | Lobular breast carcinoma (21) | -1.545 | 3.50E-02 | Cancer versus normal | Undefined on Oncomine | Zhao breast | Molecular Biology of the Cell 2004/06/01 |
| *Pkp2* | Brain (23) | Glioblastoma (81) | -2.553 | 2.05E-06 | Cancer versus normal | Human genome U133 Plus 2.0 array | Sun brain | Cancer Cell 2006/04/01 |
| *Pkp2* | Brain (23) | Oligodendroglioma (50) | -1.808 | 5.83E-04 | Cancer versus normal | Human genome U133 Plus 2.0 array | Sun brain | Cancer Cell 2006/04/01 |
| *Pkp2* | Skin (7) | Cutaneous melanoma (45) | -6.005 | 1.43E-11 | Cancer versus normal | Human genome U133A array | Talantov melanoma | Clinical Cancer Research 2005/10/15 |
| *Dsc2* | Esophageal cancer precursor (8) | Esophageal carcinoma (8) | -3.804 | 7.00E-03 | Cancer histology | Human genome U133A array | Kimchi esophagus | Cancer Research 2005/04/15 |
| *Dsc2* | Melanoma precursor (18) | Cutaneous melanoma (45) | -1.956 | 3.33E-05 | Cancer histology | Human genome U133A array | Talantov melanoma | Clinical Cancer Research 2005/10/15 |
| *Dsg2* | Melanoma precursor (18) | Cutaneous melanoma (45) | -2.777 | 4.80E-06 | Cancer histology | Human genome U133A array | Talantov melanoma | Clinical Cancer Research 2005/10/15 |
| *Dsp* | Colorectal adenoma (17) | Colorectal carcinoma (313) | -1.645 | 2.41E-04 | Cancer histology | Human genome U133A array | Bittner colon | Not published 2005/01/15 |
| *Dsp* | Esophageal cancer precursor (8) | Esophageal carcinoma (8) | -2.114 | 3.00E-03 | Cancer histology | Human genome U133A array | Kimchi esophagus | Cancer Research 2005/04/15 |
| *Dsp* | Melanoma precursor (18) | Cutaneous melanoma (45) | -51.746 | 6.20E-20 | Cancer histology | Human genome U133A array | Talantov melanoma | Clinical Cancer Research 2005/10/15 |
| *Pkp2* | Colorectal adenoma (17) | Colorectal carcinoma (313) | -1.564 | 1.00E-02 | Cancer histology | Human genome U133A array | Bittner colon | Not published 2005/01/15 |
| *Pkp2* | Esophageal cancer precursor (8) | Esophageal carcinoma (8) | -1.639 | 2.80E-02 | Cancer histology | Human genome U133A array | Kimchi esophagus | Cancer Research 2005/04/15 |
| *Pkp2* | Melanoma precursor (18) | Cutaneous melanoma (45) | -1.590 | 1.50E-02 | Cancer histology | Human genome U133A array | Talantov melanoma | Clinical Cancer Research 2005/10/15 |

^a^ Number in parentheses indicates number of samples profiled per class

^b^ Fold-change represents Class 2 as compared to Class 1

All data presented are obtained from the Oncomine online database (www.oncomine.org/resource/login.html)
