# Supplementary material for: Genetic Deletion of the Desmosomal Component Desmoplakin Promotes Tumor Microinvasion in a Mouse Model of Pancreatic Neuroendocrine Carcinogenesis
Source: PLoS Genet. 2010 Sep 16;6(9):e1001120. doi: 10.1371/journal.pgen.1001120 (PMC2940733; doi:10.1371/journal.pgen.1001120)
Supplement: Table S4 — List of primers used for quantitative PCR. (0.04 MB DOC) [file pgen.1001120.s014.doc]

**Supplemental Table 4. List of primers used for quantitative PCR.**

| **Gene symbol** | **Gene name** | **Manufacturer** | **Assay ID^a^** | **Primer/Probe Sequences^b^** |
| --- | --- | --- | --- | --- |
| *Dsc2* (mouse) | *Desmocollin 2* | Applied Biosystems | Mm01130569_m1 |  |
| *Dsg2* (mouse) | *Desmoglein 2* | Applied Biosystems | Mm00514609_m1 |  |
| *Dsp* (mouse) | *Desmoplakin* | Applied Biosystems | Mm01351874_m1 |  |
| *Pkp2* (mouse) | *Plakophilin 2* | Applied Biosystems | Mm00503159_m1 |  |
| *Cdh1* (mouse) | *Cadherin 1* | Applied Biosystems | Mm00486906_m1 |  |
| *Ctnna1* (mouse) | *Catenin alpha 1* | Applied Biosystems | Mm00486752_m1 |  |
| *Ctnnb1* (mouse) | *Catenin beta 1* | Applied Biosystems | Mm00483039_m1 |  |
| *Ctnnd1* (mouse) | *Catenin delta 1* | Applied Biosystems | Mm00483042_m1 |  |
| *Jup* (mouse) | *Junction plakoglobin* | Applied Biosystems | Mm00550249_m1 |  |
| *Igf2* (mouse) | *Insulin-like growth factor 2* | Applied Biosystems | Mm00439565_g1 |  |
| *Dsg2* (human) | *Desmoglein 2* | Applied Biosystems | Hs00170071_m1 |  |
| *Dsp* (human) | *Desmoplakin* | Applied Biosystems | Hs00189422_m1 |  |
| *Cdh1* (human) | *Cadherin 1* | Applied Biosystems | Hs00170423_m1 |  |
| *Rpl19* (mouse) | *Ribosomal protein L19* | UCSF Helen Diller Cancer Center Genome Core |  | Forward: 5’-CCAAGAAGATTGACCGCCATA-3’  Reverse: 5’-GTCAGCCAGGAGCTTCTTGC-3’  Probe: 5’-CATCCTCATGGAGCACATCCACAAGC-3’ |
| *Gusb* (human) | *Glucuronidase beta* | UCSF Helen Diller Cancer Center Genome Core |  | Forward: 5’-CTCATTTGGAATTTTGCCGATT-3’  Reverse: 5’-CCGAGTGAAGATCCCCTTTTTA-3’  Probe: 5’-TGAACAGTCACCGACGAGAGTGCTGG-3’ |

a Assay IDs are provided for primers obtained from Applied Biosystems

b Primer/probe sequences are provided for primers designed by the UCSF Helen Diller Family Cancer Center Genome Core
